# Global identification of *Arabidopsis* lncRNAs reveals the regulation of *MAF4* by a natural antisense RNA

**DOI:** 10.1038/s41467-018-07500-7

**Published:** 2018-11-29

**Authors:** Xinyue Zhao, Jingrui Li, Bi Lian, Hanqing Gu, Yan Li, Yijun Qi

**Affiliations:** 1 Center for Plant Biology, School of Life Sciences, Tsinghua University, Beijing, 100084 China; 2 Tsinghua-Peking Center for Life Sciences, Beijing, 100084 China

## Abstract

Long non-coding RNAs (lncRNAs) have emerged as important regulators of gene expression and plant development. Here, we identified 6,510 lncRNAs in *Arabidopsis* under normal or stress conditions. We found that the expression of natural antisense transcripts (NATs) that are transcribed in the opposite direction of protein-coding genes often positively correlates with and is required for the expression of their cognate sense genes. We further characterized *MAS*, a NAT-lncRNA produced from the *MADS AFFECTING FLOWERING4* (*MAF4)* locus. *MAS* is induced by cold and indispensable for the activation of *MAF4* transcription and suppression of precocious flowering. *MAS* activates *MAF4* by interacting with WDR5a, one core component of the COMPASS-like complexes, and recruiting WDR5a to *MAF4* to enhance histone 3 lysine 4 trimethylation (H3K4me3). Our study greatly extends the repertoire of lncRNAs in *Arabidopsis* and reveals a role for NAT-lncRNAs in regulating gene expression in vernalization response and likely in other biological processes.

## Abstract (web summary)

Long non-coding RNAs regulate developmental transitions and stress responses in plants. Here Zhao et al. show that a non-coding antisense transcript *MAS* transcribed from the *Arabidopsis MAF4* locus activates H3K4me3 deposition and *MAF4* transcription to suppress precocious flowering.

## Introduction

Long non-coding RNAs (lncRNAs) have emerged as important players in the regulation of gene transcription, splicing, and translation^1,2^. Based on their relationship with protein-coding genes, lncRNAs can be classified as natural antisense transcripts (NATs), overlapping lncRNAs (OT-lncRNAs), long intergenic non-coding RNAs (lincRNAs), and intronic non-coding RNAs (incRNAs)^3,4^. NAT-lncRNAs are transcribed in the opposite direction of protein-coding genes, OT-lncRNAs partially or fully overlap protein-coding genes in the sense direction, whereas lincRNAs and incRNAs originate from intergenic and intronic regions, respectively.

NAT-lncRNAs are widespread in animals^5–8^ and plants^9–12^. They and their cognate sense transcripts often exhibit concordant or discordant expression patterns^7,13^. NAT-lncRNAs may positively or negatively regulate the expression of their sense transcripts using diverse transcriptional or post-transcriptional mechanisms. The transcriptional machineries of NAT-lncRNAs and their sense transcripts may compete for RNA Polymerase II (RNA Pol II) and regulatory transcription factors, or undergo collision, resulting in transcriptional interference^5,14,15^. Moreover, NAT-lncRNAs can serve as scaffolds to recruit DNA-modifying and histone-modifying enzymes, thereby facilitating DNA methylation, histone modifications, chromatin conformation changes, and eventually upregulation or downregulation of gene transcription^5,14–17^. Post-transcriptionally, NAT-lncRNAs may affect mRNA decay by nucleases, mask miRNA binding sites, modulate protein translation or produce endogenous siRNAs to execute RNA interference (RNAi)^1,5,14,18^.

In plants, thousands of lncRNAs have been identified and implicated in root development, seedling light response, flowering time control, reproduction, and stress response^11,12,19–28^. However, only a handful of plant lncRNAs have been experimentally characterized. *COOLAIR* is a set of alternatively spliced and polyadenylated transcripts transcribed from the *FLOWERING LOCUS C (FLC)* locus at an early stage of cold exposure^29,30^ and mediates the reduction of active histone mark H3 lysine 36 trimethylation (H3K36me3) and an increase of repressive histone mark H3K27me3^31^. *COLDAIR* is induced at a later stage of cold exposure and cooperates with an *FLC* promoter-derived lncRNA *COLDWRAP* to establish high H3K27me3 and silence *FLC*^32,33^. The lincRNA *APOLO* is transcribed in response to auxin and regulates root development through mediating the formation of a chromatin loop encompassing the promoter of its neighboring gene *PID* and downregulating the transcription of *PID*^19,34^. The lncRNA *HID1* induced by continuous red light also transcriptionally suppresses its target gene and promotes seedling photomorphogenesis^35^. The elf18-induced lncRNA *ELENA* enhances *PR1* expression through interacting with MED19a and affecting its enrichment on the *PR1* promoter^36^. Instead of being transcriptional regulators, *ASCO-lncRNA* was found to associate with the nuclear speckle RNA-binding protein (NSR) and modulate NSR-mediated alternative splicing events through mimicking and displacing pre-mRNA targets^37^. Similarly, the lncRNA *IPS1* inhibits the activity of phosphate starvation-induced miR399 by mimicking and sequestering miR399 target mRNA^38^. Two rice lncRNAs *PMS1T* and *LDMAR* were shown to regulate photoperiod-sensitive male sterility^39,40^. Whereas *PMS1T* functions through generating phased small interfering RNAs (phasiRNAs)^41^, the molecular basis of *LDMAR* function remains a mystery.

In this study, in order to explore the function of lncRNAs in gene regulation and the range of such regulation in plants, we employed high-depth strand-specific RNA sequencing (RNA-seq) to systematically identify lncRNAs in *Arabidopsis thaliana*. We annotated 6510 lncRNAs including 4050 NAT-lncRNAs and 2460 lincRNAs. We found that many NAT-lncRNAs and their cognate protein-coding sense transcripts are concordantly expressed in different tissues or under stress conditions and knocking down NAT-lncRNAs leads to decreased expression of sense transcripts. We further demonstrated that one NAT-lncRNA, *MAS*, positively regulates the transcription of its cognate sense gene *MAF4* through interacting with and recruiting WDR5a, a core component of the COMPASS-like complexes, to *MAF4*, thereby regulating flowering time. Our study provides a resource for studying lncRNAs in *Arabidopsis* and reveals a mechanism for gene regulation by NAT-lncRNAs.

## Results

### Global identification of lncRNAs in *Arabidopsis*

**Fig. 1:**
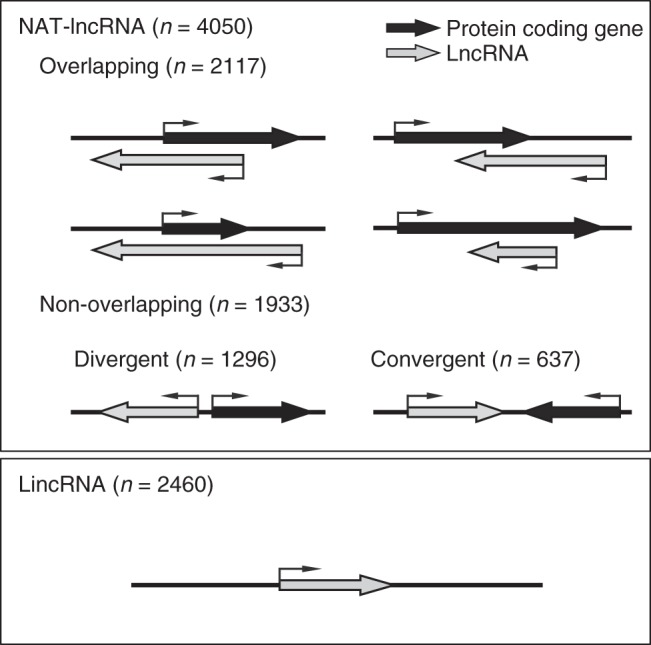
Annotation of lncRNAs in *Arabidopsis*. LncRNAs are classified into two categories based on their genomic locations: NAT-lncRNAs and lincRNAs. NAT-lncRNAs include overlapping NAT-lncRNAs and non-overlapping NAT-lncRNAs. Non-coding and coding transcripts are depicted as gray and black bars, respectively. Arrows indicate the directions of transcription

To globally identify lncRNAs in *Arabidopsis*, we reconstructed an *Arabidopsis* transcriptome using high-depth strand-specific RNA sequencing (ssRNA-seq). We generated cDNA libraries for rRNA-depleted total, polyadenylated [poly(A)+] and non-polyadenylated [poly(A)−] RNAs in whole cell extract, nuclear and cytosolic fractions that were prepared from *Arabidopsis* grown under normal or stress conditions (Supplementary Data 1, RNA-seq datasets numbered 1–34). A total of 1.2 billion genome-matched reads were obtained. These reads, together with the reads obtained from 3 published RNA-seq datasets^11^, were assembled to reconstruct the *Arabidopsis* transcriptome. This resulted in 106,421 unique transcripts from 64,987 genomic loci. Among these, 25,245 were previously annotated protein-coding transcripts (TAIR10), accounting for 93% of all annotated protein-coding transcripts. This indicates that the reconstructed transcriptome had reasonably high coverage and quality. After the removal of 39,082 transcripts corresponding to protein-coding transcripts, other known ncRNAs (e.g., miRNAs, tRNAs, and rRNAs), 29,463 transcripts with short length (< 150 nt) or low abundance (FPKM_MAX_ < 1), 25,270 transcripts with protein-coding potential (CPC score > 0), and 6096 transcripts partially or fully overlapping with protein-coding genes in the sense direction, we annotated 6510 lncRNAs ((Supplementary Fig. 1a and Supplementary Data 2). These lncRNAs include 4050 NAT-lncRNAs and 2460 lincRNAs (Fig.1). NAT-lncRNAs were further classified into overlapping (2117), divergent (1296) and convergent (637) NAT-lncRNAs (Fig. 1).

### Characteristics of *Arabidopsis* lncRNAs

We analyzed features of the identified lncRNAs including average size, exon number, isoform number, and expression level. Same analyses were also performed for protein-coding transcripts in parallel for comparison. We found that lncRNAs were much shorter than coding RNAs (mean length of 633 nt for lncRNAs versus 1408 nt for coding RNAs) (*P*-value < 0.0001, Mann–Whitney *U*-Test, one-tailed) (Supplementary Fig. 1b). The lncRNAs had fewer exons (mean = 3.7) than coding RNAs (mean = 5.9) (*P*-value < 0.0001, Mann–Whitney *U*-Test, one-tailed) (Supplementary Fig. 1c) and smaller number of isoforms (mean = 1.3) comparable to coding RNAs (mean = 1.4) (Supplementary Fig. 1d). The expression levels of lncRNAs and coding RNAs were estimated by fragments per kb of exonic sequence per million mapped reads (FPKM) using Cuffdiff^42^. The expression levels of lncRNAs were lower than those of coding RNAs (*P*-value < 0.0001, Mann–Whitney *U*-Test, one-tailed) (Supplementary Fig. 1e).

We examined whether lncRNAs are polyadenylated, taking advantage of RNA-seq datasets for poly(A)+ [SW_poly(A) +] and poly(A)− [SW_poly(A)−] RNAs (Supplementary Data 1). By applying a strict criterion (*P*-value < 0.05 and fold-change ≥2), we found that 1352 lncRNAs were significantly enriched in the poly(A)+ fraction, whereas 198 lncRNAs were significantly enriched in the poly(A)− fraction (Supplementary Fig. 2a and Supplementary Data 3). The presence or absence of poly(A) in representative lncRNAs was validated by RT-PCR analyses (Supplementary Fig. 2b).

We estimated the partitioning of each lncRNA between the nucleus and the cytoplasm by analyzing the RNA-seq datasets for cytosolic (SC_Total) and nuclear fractions (SN_Total) (Supplementary Data 1). We found that 239 lncRNAs had significantly higher levels in the nuclear fraction than that in the cytosolic fraction, whereas only 43 lncRNAs were more abundant in the cytosolic fraction (*P*-value < 0.05 and fold-change ≥2) (Supplementary Fig. 2c and Supplementary Data 4). RT-PCR analyses with fractionated nuclear and cytosolic extracts confirmed that all 10 randomly selected lncRNAs were predominantly localized in the nucleus (Supplementary Fig. 2d).

### LncRNAs are developmentally and physiologically regulated

**Fig. 2:**
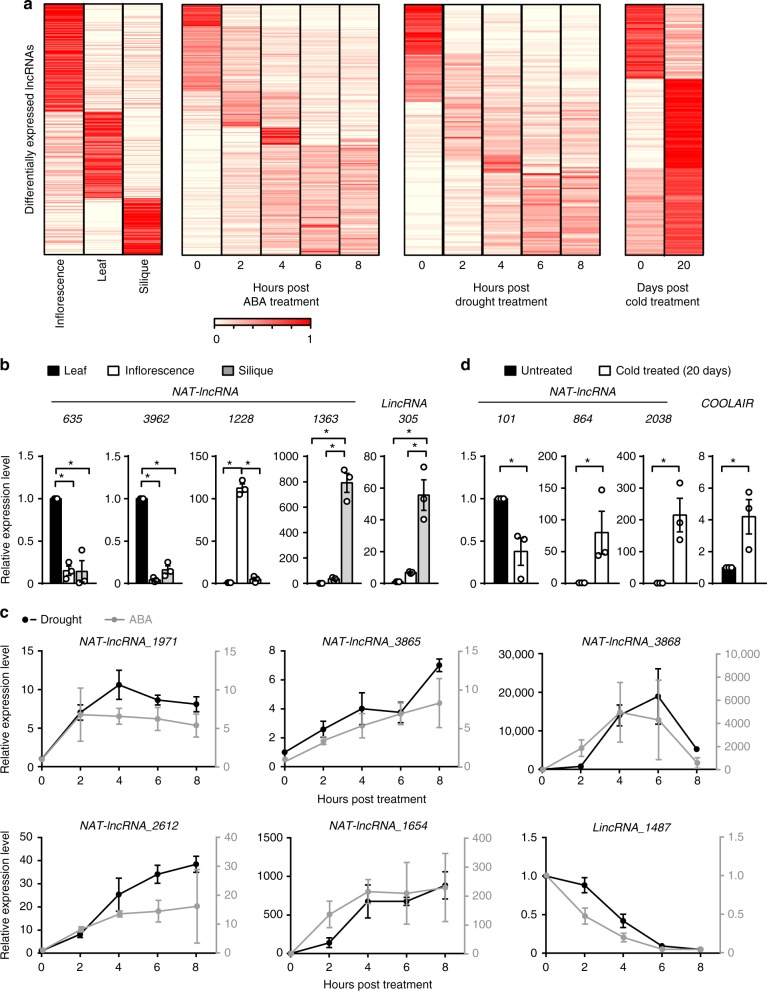
LncRNAs are developmentally and physiologically regulated. **a** Heat maps showing the abundances of differentially expressed lncRNAs in different plant tissues and in plants treated with ABA, drought or cold. Rows are ordered based on a k-means clustering of lncRNAs. Color intensity represents the fractional density across the row of FPKM counts. **b**–**d** Detection of representative lncRNAs in the indicated samples by RT-qPCR. Error bars represent s.e.m (*n* = 3), asterisks indicate a significant difference (*t-*test, *P*-value < 0.05). Source data are provided as a Source Data file

To investigate whether the identified lncRNAs are developmentally and physiologically regulated, we estimated the expression levels of each lncRNA by calculating FPKM in different tissues (seedling, inflorescence, and siliques) or under different treatments (cold, ABA and drought) using the RNA-seq datasets, which include three biological replicates for each sample. The Pearson correlation coefficients close to 1 indicate high reproducibility of the RNA-seq experiments (Supplementary Fig. 3). We found that 627 lncRNAs had differential expression in different tissues (*P-*value < 0.05 and fold-change ≥2) (Fig. 2a and Supplementary Data 5). 510 and 509 lncRNAs showed inducible expression patterns at one time point upon ABA and drought treatment, respectively (Fig. 2a and Supplementary Data 6, 7). We also found that 196 lncRNAs including *COOLAIR* showed a significant increase or decrease in their expression levels after cold treatment (Fig. 2a and Supplementary Data 8). The expression patterns of several randomly selected lncRNAs were confirmed by quantitative RT-PCR (RT-qPCR) (Fig. 2b-d). These data show the dynamic changes of lncRNA expression in response to developmental and environmental cues and suggest their roles in development and stress responses.

### NAT-lncRNAs regulate the expression of cognate sense genes

**Fig. 3:**
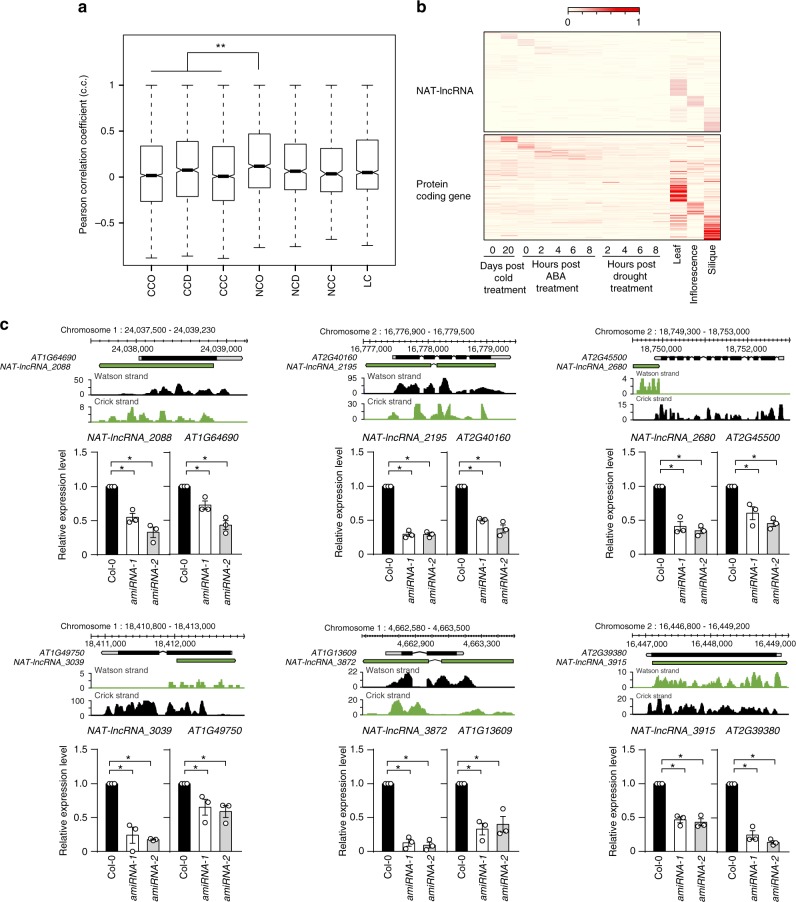
NAT-lncRNAs regulate the expression of cognate sense genes. **a** A boxplot showing the correlation of expression patterns between neighboring gene pairs. CCO, overlapped protein-coding gene pair; CCD, divergent protein-coding gene pair; CCC, convergent protein-coding gene pair; NCO, overlapping NAT-lncRNA and associated protein-coding gene pair; NCD, divergent NAT-lncRNA and closest neighboring gene pair; NCC, convergent NAT-lncRNA and closest neighboring gene pair; LC, lincRNA and closest neighboring gene pair. The central lines, bounds of box represent the median, 25% quartile and 75% quartile. The whiskers represent 1.5 × IQR of the lower or upper quartile. Asterisks indicate a significant difference between the indicated groups (Mann–Whitney *U-*test, *P-*value < 0.01). **b** Heat maps showing the expression patterns of 216 NCO pairs. Rows are ordered based on a k-means clustering of NAT-lncRNAs. Color intensity represents the fractional density across the row of FPKM counts. **c** Detection of NATs-lncRNAs and their cognate sense genes by RT-qPCR in Col-0 and indicated *amiRNA* knockdown lines. Error bars represent s.e.m (*n* = 3), asterisks indicate a significant difference (*t*-test, *P-*value < 0.05). Source data are provided as a Source Data file. Shown above the RT-qPCR results are genome browser views of RNA-seq signals at NAT-lncRNAs and cognate sense genes in Col-0, with normalized read counts per million along the y-axis. More examples are available in Supplementary Figs. 5, 6

To explore the function of lncRNAs in gene regulation, we first examined whether lncRNAs and their adjacent genes are concordantly or discordantly expressed. We calculated the Pearson correlation coefficients (p.c.c.) between the different types of lncRNAs and their adjacent protein-coding genes. The p.c.c. values between adjacent protein-coding gene pairs were calculated in parallel for comparison. We found that the p.c.c. values of overlapping NAT-lncRNA/sense gene pairs were significantly higher than the values between adjacent protein-coding pairs (Fig. 3a), suggesting that overlapping NAT-lncRNAs have a stronger tendency to have positively correlated expression patterns with their sense overlapping genes. The concordant expression patterns of 216 overlapping NAT-lncRNAs and their cognate sense genes (p.c.c. score > 0.6) are shown in Fig. 3b.

The finding of concordant expression of NAT-lncRNAs and their cognate genes led us to examine whether NAT-lncRNAs play a role in regulating the expression of their cognate genes. We knocked down 21 NAT-lncRNAs using artificial microRNAs (amiRNAs) (Supplementary Fig. 4). Interestingly, the reduction of 15 and 3 NAT-lncRNAs resulted in significantly decreased and increased expression of their cognate sense genes, respectively. The reduction of other 3 NAT-lncRNAs did not significantly change the expression of their cognate sense genes (Fig. 3c, Supplementary Figs. 5, 6). Alteration of sense gene expression in *amiRNA* knockdown lines was not due to targeting of sense genes by amiRNA*s. Eight out of 21 amiRNA*s do not base pair with sense mRNAs at all. The rest of the amiRNA*s have mismatches to corresponding sense mRNAs at critical positions (Supplementary Fig. 4). Furthermore, most of the amiRNA*s do not have 5’ terminal uridine (Supplementary Fig. 4), making it less likely that they are loaded into the effector AGO1 to suppress gene expression^43^. To further rule out the possibility that production of secondary siRNAs targeting sense genes leads to alteration of sense gene expression, we performed small RNA (sRNA) sequencing on 12 randomly chosen *amiRNA* knockdown lines. The results revealed that no secondary siRNAs were detected in these lines (Supplementary Fig. 7). Together, our data suggest that NAT-lncRNAs are involved in the regulation of cognate sense gene expression.

### A natural antisense lncRNA regulates *MAF4* gene expression

**Fig. 4:**
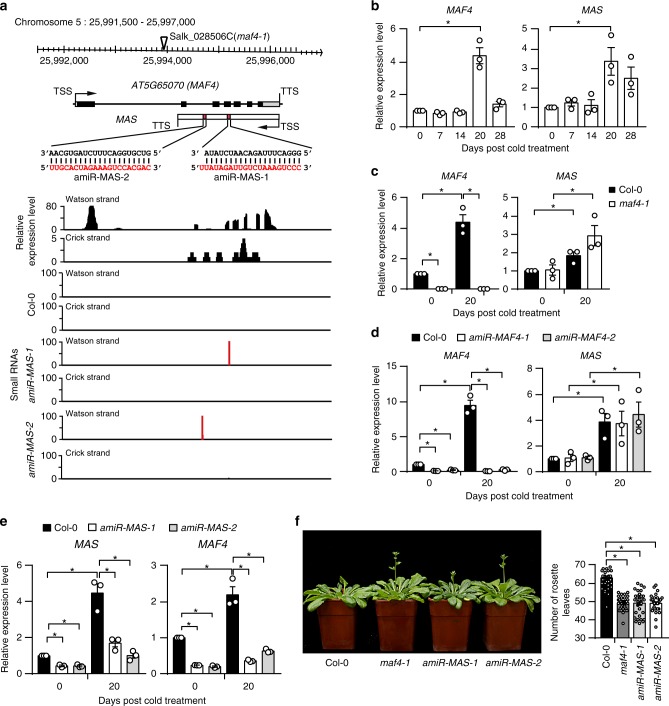
A natural antisense lncRNA regulates *MAF4* gene expression. **a** Genome browser view of *MAF4* and *MAS*. Signals from RNA- and sRNA-seq are shown, with normalized read counts per million along the y-axis. T-DNA insertion and amiRNA target sites are indicated. TSS, transcription start site; TTS, transcription termination site. **b** Detection of *MAF4* and *MAS* in Col-0 after 0–28 d of cold exposure by RT-qPCR. **c** Detection of *MAF4* and *MAS* in Col-0 and *maf4-1* before (0 d) and after 20 d of cold exposure. **d** Detection of *MAF4* and *MAS* in Col-0 and two *MAF4* amiRNA knockdown lines (*amiR-MAF4-1* and *amiR-MAF4-2*) before (0 d) and after 20 d of cold exposure. **e** Detection of *MAS* and *MAF4* in Col-0 and two *MAS* amiRNA knockdown lines (*amiR-MAS-1* and *amiR-MAS-2*) before (0 d) and after 20 d of cold exposure. In **b**–**e**, error bars represent s.e.m (*n* = 3), asterisks indicate a significant difference between the indicated groups (*t-*test, *P-*value < 0.05). **f** Flowering-time phenotypes of Col-0, *maf4-1*, and *amiR-MAS-1/2* lines grown in SD conditions after 20 d of cold exposure. Numbers of primary rosette leaves were counted when bolts were ~ 3–5 cm long. At least thirty plants of each genotype were used for statistical analysis. Asterisks indicate a significant difference (*t-*test, *P-*value < 0.05). Source data are provided as a Source Data file

The finding that NAT-lncRNAs regulates cognate sense gene expression prompted us to investigate the biological importance of such regulation. We focused on one NAT-lncRNA, *NAT-lncRNA_2962*. *NAT-lncRNA_2962* is transcribed from the antisense strand of the cold-responsive *MAF4* gene, a *FLC* family member that functions to prevent precocious vernalization response^44–46^. We renamed it *MAS* for *MAF4* antisense RNA (Fig. 4a). RACE analyses showed that the 5’ end of *MAS* is initiated at a site several nucleotides to the transcription termination site (TTS) of *MAF4* and the 3’ end of *MAS* extends into the 1st intron of *MAF4* and undergoes polyadenylation (Supplementary Fig. 8a).

*MAF4* is induced during early periods of cold exposure and its expression peaks at 20th day of cold exposure^44^. We validated the temporal expression pattern of *MAF4* by RT-qPCR. Intriguingly, the expression pattern of *MAS* during cold treatment closely mimicked that of *MAF4* (Fig. 4b). The concordant expression of *MAF4* and *MAS* suggests that either *MAF4* transcript promotes *MAS* expression or vice versa. We tested the first possibility by examining the expression of *MAS* in *maf4-1*^44^ that contains a T-DNA insertion in the largest intron of *MAF4* and has abolished *MAF4* expression before and after cold treatment (Fig. 4c) and two *amiRNA* lines (*amiR-MAF4-1* and *amiR-MAF4-2*) in which *MAF4* transcript was knocked down (Fig. 4d). The basal expression and induction of *MAS* were not disturbed in both *maf4-1* and *amiR-MAF4-1/2* (Fig. 4c, d), indicating that *MAF4* transcript does not affect *MAS* expression. We then tested whether *MAS* regulates *MAF4* expression. We generated two *amiRNA* lines (*amiR-MAS-1* and *amiR-MAS-2*) in which *MAS* transcript was knocked down (Fig. 4a, e). In both lines, the basal level of *MAF4* transcript was reduced and the induction of *MAF4* expression by cold was severely compromised as well (Fig. 4e). Similar to the reduction of sense gene expression in other *amiRNA* knockdown lines we generated (Supplementary Fig. 4), the reduction of *MAF4* expression was not due to amiRNA*s targeting *MAF4* mRNA (Supplementary Fig. 8b) or amiRNA-triggered production of secondary siRNAs (Fig. 4a). Thus, our data strongly support the notion that *MAS* plays a positive role in *MAF4* expression. *MAF5*^44–46^, another *FLC* family member that functions to prevent precocious vernalization response, is near the *MAF4* gene. *MAF5* expression remained unaltered in *amiR-MAS-1/2*, suggesting that *MAS* does not regulate the expression of *MAF5* (Supplementary Fig. 8c).

The role of *MAS* in *MAF4* expression prompted us to examine whether *MAS* also acts as a floral repressor. We examined the flowering phenotype of *amiR-MAS-1/2*, *maf4-1* and the wild-type (Col-0) plants grown in short-day conditions after 20 days of cold exposure. We found that, like the *maf4-1* mutant, a*miR-MAS1/2* flowered earlier than Col-0 (Fig. 4f). All together, these results suggest that *MAS* transcript is necessary for the expression of *MAF4* and repression of flowering.

### *MAS* promotes *MAF4* expression at the transcriptional level

We next investigated how *MAS* regulates *MAF4*. As *MAS* is complementary to the *MAF4* transcript, it was possible that they form a double-stranded RNA to produce sRNAs. However, few sRNAs were detected at the overlapping region of *MAS* and *MAF4* (Fig. 4a)^47^, excluding the possibility that *MAS* regulates *MAF4* via a mechanism involving sRNAs.

**Fig. 5:**
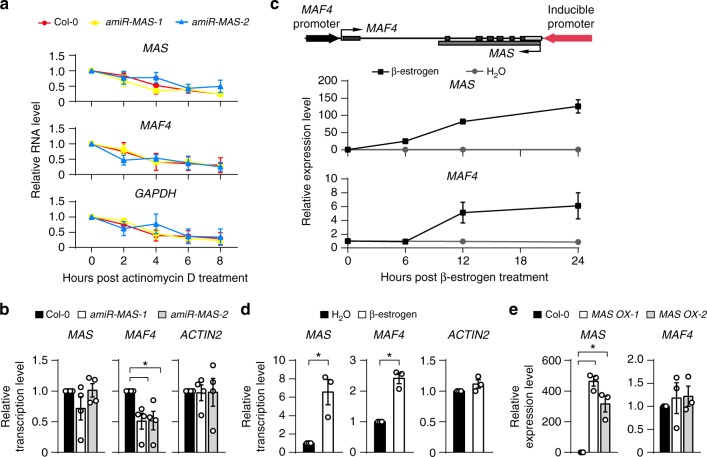
MAS regulates *MAF4* at the transcriptional level. **a** Remaining levels of *MAS*, *MAF4*, and *GAPDH* at different time points post actinomycin D (100 μg/mL) treatment in Col-0 and *amiR-MAS-1/2* lines. Plants were cold-treated for 20 d prior to actinomycin D treatment. **b** Transcription rates of *MAS*, *MAF4*, and *ACTIN2* measured by nuclear run-on assay and RT-qPCR in Col-0 and *amiR-MAS-1/2* lines after 20 d of cold exposure. **c** Upper panel, schematic representation of the construct used to generate the transgenic line expressing *MAS* under the control of a β-estrogen-inducible promoter. Lower panel, time-course of β-estradiol (20 μM)-inducible *MAS* and *MAF4* expression as determined by RT-qPCR. **d** Transcription rates of *MAS*, *MAF4,* and *ACTIN2* as measured by nuclear run-on assay in the transgenic line treated with β-estradiol for 24 h. **e** Detection of *MAS* and *MAF4* in Col-0 and two transgenic lines overexpressing *MAS* (*MAS OX-1* and *MAS OX-2*). In **b**–**e**, error bars represent s.e.m (*n* = 3-4), asterisks indicate a significant difference (*t*-test, *P*-value < 0.05). Source data are provided as a Source Data file

To examine whether *MAS* regulates *MAF4* expression through modulating the stability of *MAF4* transcripts, we measured the RNA decay rate of *MAF4* in Col-0 and *amiR-MAS-1/2* lines treated with the transcriptional inhibitor actinomycin D (ActD). ActD effectively blocked the transcription of both *MAF4* and *MAS* as well as that of *GAPDH*. However, the decline rates of *MAF4* transcripts in Col-0 and *amiR-MAS-1/2* were indistinguishable, suggesting that *MAS* does not regulate the stability of *MAF4* transcript (Fig. 5a).

We next tested whether *MAS* transcriptionally promotes *MAF4* expression. We performed nuclear run-on (NRO) assay to assess the transcriptional rate of *MAF4* in Col-0 and *amiR-MAS-1/2*. Knockdown of *MAS* transcript caused a significant reduction of *MAF4* transcriptional rate, but not that of *ACTIN2* or *MAS* itself, indicating that *MAS* controls *MAF4* expression at the transcriptional level (Fig. 5b). To further demonstrate that *MAS* expression can transcriptionally activate *MAF4*, we generated a transgenic line in which the genomic sequence of *MAF4* (including the promoter and coding region of *MAF4*) was reversely fused to a β-estrogen-inducible promoter^48^ that drives the expression of *MAS* (Fig. 5c). As expected, β-estrogen treatment induced the transcription of *MAS*, and more importantly, the transcription of *MAF4* was also induced (Fig. 5c, d).

We asked whether *MAF4* could be regulated by ectopically expressed *MAS*. We then generated two transgenic lines (*MASOX-1* and *MASOX-2*) in which *MAS* transcript was overexpressed most likely at loci other than *MAF4* (Fig. 5e*)*. The expression level of *MAF4* did not change in either transgenic line (Fig. 5e), suggesting that *MAS* functions to regulate *MAF4* in *cis*, but not in *trans*. Taken together, our data indicate that cis-acting *MAS* activates *MAF4* expression at the transcriptional level.

### *MAS* mediates the recruitment of WDR5a to *MAF4*

**Fig. 6:**
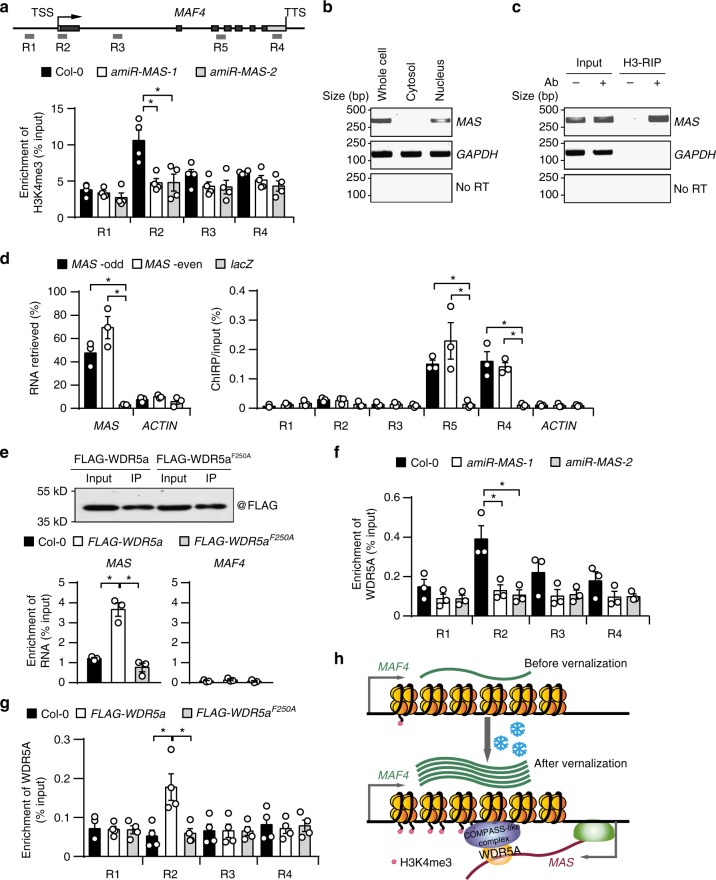
*MAS* mediates the recruitment of WDR5a to *MAF4*. **a** Upper panel, schematic representation of *MAF4* locus, the positions of primers (R1 to R5) used for ChIP- and ChIRP-qPCR are indicated. Lower panel, detection of H3K4me3 levels in Col-0 and *amiR-MAS-1/2* lines after 20 d of cold exposure by ChIP-qPCR. **b** Detection of *MAS* and *GAPDH* in the whole cell, cytoplasmic and nuclear fractions by RT-PCR. **c** Detection of *MAS* and *GAPDH* in histone 3 (H3) immunoprecipitates by RT-PCR. **d** ChIRP-qPCR analyses of *MAS* association with *MAF4* locus after 20 d of cold exposure. Left panel, ChIRP enrichment of *MAS* transcript, but not *Actin* transcript in both odd and even probe pools. Right panel, qPCR detection of different regions of *MAF4* locus in immunoprecipitated DNA. Probes targeting *LacZ* mRNA were used as negative controls. **e** Association between *MAS* and WDR5a detected by RIP with anti-FLAG Magnetic Beads in control and transgenic (*FLAG-WDR5a* and *FLAG-WDR5a*^*F250A*^) plants after 20 d of cold exposure. Purification of WDR5a and WDR5a^F250A^ was validated by western blot (upper panel). The levels of *MAS* and *MAF4* in the immunoprecipitates were determined by RT-qPCR (lower panel). **f** Detection of WDR5a at *MAF4* locus by ChIP-qPCR with an antibody against WDR5 in Col-0 and *amiR-MAS-1/2* lines after 20 d of cold exposure. **g** Detection of WDR5a and WDR5a^F250A^ at *MAF4* locus by ChIP-qPCR in Col-0 and the transgenic plants after 20 d of cold exposure. In (**a**), **d**–**g** error bars represent s.e.m (*n* = 3–4), asterisks indicate a significant difference between the indicated groups (*t-*test, *P-*value < 0.05). **h** A model for *MAS*-mediated activation of *MAF4* gene expression during vernalization. Source data are provided as a Source Data file

In *Arabidopsis*, H3K4me3 has been implicated in transcriptional activation of genes^49^, including *MAF4*^44,46^. To explore whether *MAS* mediates *MAF4* gene activation through regulating H3K4me3 deposition, we detected H3K4me3 levels at the *MAF4* locus in Col-0 and *amiR-MAS-1/2* lines. We found that H3K4me3 was highly enriched at the transcription start site (TSS) of the *MAF4* locus in Col-0; however, such enrichment was significantly reduced in *amiR-MAS-1/2* (Fig. 6a), suggesting that *MAS* plays a role in enhancing H3K4me3 deposition at *MAF4*. We also detected the levels of active marks H3K27Ac and H3K36me3, and the repressive mark H3K27me3 at the *MAF4* locus in Col-0 and *amiR-MAS-1/2*. Interestingly, the levels of H3K27Ac and H3K36me3 remained unaltered while the levels of H3K27me3 were slightly increased in *amiR-MAS-1/2* (Supplementary Fig. 9).

H3K4me3 is conservatively catalyzed by the WDR5/MLL complexes (also called COMPASS-like complexes in *Arabidopsis*)^50,51^. WDR5a is a plant homolog of human WDR5 (Supplementary Fig. 10a). We used *WDR5a* RNAi lines^51^ to determine whether *WDR5a* is essential for H3K4me3 deposition at *MAF4* and its activation. We found that *WDR5a* knockdown resulted in a great reduction of H3K4me3 level at *MAF4* (Supplementary Fig. 10b) and impaired cold-induced *MAF4* expression (Supplementary Fig. 10c), suggesting that WDR5a is required for H3K4me3 deposition and activation of *MAF4*.

In mammals, lncRNAs are involved in targeting WDR5/MLL complexes to specific loci through interacting with WDR5^52–54^. We thus examined whether *MAS* associates with WDR5a to guide WDR5a to *MAF4*. We first confirmed that *MAS* transcript was retained in the nucleus and associated with the chromatin fraction (Fig. 6b, c). We further carried out chromatin isolation by RNA purification coupled with qPCR (ChIRP-qPCR) and found that *MAS* bound the *MAF4* gene (Fig. 6d). We next generated transgenic lines expressing FLAG-tagged WDR5a or WDR5a^F250A^, a mutant form of WDR5 lacking RNA binding capability^54^. We immunoprecipitated FLAG-tagged WDR5a and WDR5a^F250A^ from cold-treated transgenic plants and detected *MAS* transcript in the immunoprecipitates. We found that WDR5a but not WDR5a^F250A^ pulled down *MAS* (Fig. 6e). In contrast to the case of *MAS*, neither WDR5a nor WDR5a^F250A^ associated with *MAF4* transcript (Fig. 6e).

To further determine the role of *MAS* in targeting WDR5a to *MAF4*, we detected the enrichment of WDR5a at *MAF4* in Col-0 and *amiR-MAS-1/2* lines. *MAS* knockdown greatly reduced the enrichment of WDR5a at *MAF4* (Fig. 6f). Moreover, WDR5a, but not WDR5a^F250A^, could accumulate at *MAF4* (Fig. 6g), suggesting that *MAS* binding is important for WDR5a recruitment.

Collectively, our data suggest that *MAS* is transcribed during cold exposure and its induction plays a crucial role in the recruitment of WDR5a to *MAF4* to activate the expression of *MAF4* (Fig. 6h).

## Discussion

The function and range of lncRNA-mediated regulation in plants have been increasingly appreciated. In this study, we identified a large number of lncRNAs and analyzed their expression profile in different tissues under normal or stress conditions using strand-specific RNA-seq. We sequenced poly(A)+ and poly(A)−, nuclear and cytoplasmic RNAs separately to increase the sensitivity of detecting lncRNAs with distinct features. Indeed, ~ 88% of the lncRNAs we identified have not been previously discovered by tilling arrays or conventional RNA-seq^11,20,23^. Thus, the lncRNAs we identified represent a valuable addition to the *Arabidopsis* lncRNA collection, and provide a rich resource for the community to further investigate the biology of lncRNAs in plants.

We focused on the function of a NAT-lncRNA *MAS* in the activation of *MAF4* expression during cold treatment. We found that that *MAS* acts in *cis* to activate *MAF4* expression at the transcriptional level (Figs. 4, 5). The transcriptional activating role of *MAS* is similar to that played by lncRNAs *HOTTIP*^53^, *NeST*^52^, *LAIR*^55^, and *EVX1as*^56^. However, the mechanisms adopted by these lncRNAs are varied. *MAS* binds WDR5a and then guides the COMPASS-like complexes to *MAF4* to promote H3K4me3 (Fig. 6). Like *MAS*, *HOTTIP* also interacts with WDR5 and recruits the MLL complex to maintain H3K4me3 and activation of *HOXA* genes^53^. However, the cis-regulatory action of *HOTTIP* and *NeST* requires the chromosome looping that brings the *HOTTIP* or *NeST* locus into close spatial proximity to its target genes^53^. Ectopic overexpression of *MAS* cannot stimulate *MAF4* expression, whereas ectopic overexpression of *LAIR* promotes the upregulation of *LRK* genes. *EVX1as* increases the transcription of *EVX1* through facilitating the binding of Mediator complex to *EVX1* region, leading to an active chromatin state.

Exemplified by *MAS*, many NAT-lncRNAs were found to be concordantly expressed with their sense genes (Fig. 3), suggesting co-upregulation of NAT-lncRNAs and their sense genes. Our results are consistent with previous findings that neighboring genes often have correlated expression irrespective of their orientations^57^. Also, previous study of an immediate-early gene (IEGs) revealed that the ripple effect plays an important role in transcriptional activation of IEGs and their neighboring genes^58^. However, the cases of NAT-lncRNAs and comparisons between the ripple effects triggered by lncRNAs and regular genes were not included in the previous analysis^58^. Our genome-wide analysis revealed that NAT-lncRNAs are significantly more likely to produce ripple effects and activate their sense overlapping genes than regular genes and other types of lncRNAs (Fig. 3a). On one hand, this could be because the average distances between the TSSs of NAT-lncRNAs and their paired genes are smaller. On the other hand, this may reflect the fact that NAT-lncRNAs play crucial roles in activating the expression of their paired genes. We found that some NAT-lncRNAs are indeed required for the expression of their sense overlapping genes, suggesting that this cis-regulatory mode could be common to many NAT-lncRNAs (Fig. 3c and Supplementary Figs. 3, 4). Whether these NAT-lncRNAs regulate their cognate sense genes through recruiting the COMPASS-like complexes or other mechanisms remains to be investigated.

Our finding that many lncRNAs are responsive to different stresses suggests that plant lncRNAs may play crucial biological roles. *COOLAIR* and *COLDAIR* have been found to mediate vernalization-induced repression of the floral repressor *FLC*^29,32^. Here we demonstrate that the lncRNA *MAS*, by regulating the expression of an *FLC* family member, *MAF4*, fine-tunes the time of flowering. However, different from the repressive roles of *COOLAIR* and *COLDAIR*, *MAS* activates the expression of *MAF4*. Whereas *COLDAIR* associates with a subunit of the conserved repressive complex PRC2^32^, *MAS* binds to the core component of the COMPASS-like complex that achieves transcriptional activation. Then why the floral repressor *FLC* and *MAF4* are oppositely regulated upon cold exposure? It was suggested that *MAF4* and *MAF5* are transiently activated to prevent precocious flowering so that plants only flower after a long period of cold when *FLC* is completely silenced^44^. The dynamic and different expression profiles of *FLC* and *MAF4* highlight the important role of lncRNAs in coordinating the vernalization response. However, the majority of lncRNAs, involved in flowering time control or other stress responses, are still awaiting functional characterization.

## Methods

### Plant materials and growth conditions

All plants used in this study are in the Col-0 background. Detailed information about mutants and generation of transgenic plants can be found in Supplementary Methods. Plants were grown on 1/2 MS medium with 30 g/L sucrose in long-day (LD, 16 h light, 22 °C / 8 h dark, 18 °C) or short-day conditions (SD, 8 h light, 22 °C / 16 h dark, 18 °C).

Stress treatments were performed as previously described with some modifications^44,59–61^. For ABA treatment, 2-week-old seedlings were transferred to 1/2 MS liquid medium with 100 μM ABA. For dehydration treatment, 2-week-old seedlings were removed from the agar and desiccated in dishes. After being treated for different time periods (0, 2, 4, 6, 8 h), the plants were harvested for RNA isolation. For cold treatment, 2-week-old seedlings (grown under SD conditions) were transferred to 4 °C and cultured under SD conditions for different time periods. After treatment, the plants were harvested or transplanted into soil and grown under SD conditions for flowering time test.

### Nuclear and cytosolic fractionation

Nuclear and cytosolic fractionation was performed as previously described^62^. The plant tissues were ground into fine powder. For cytosolic fraction, 2 volumes of lysis buffer (20 mM Tris-HCl pH 7.4, 25% glycerol, 20 mM KCl, 2 mM EDTA, 2.5 mM MgCl_2_, 250 mM sucrose, 5 mM DTT, 40 U/mL RNase inhibitor) were added to the powder. After filtration and centrifugation at 13,000 × *g* for 10 min at 4 °C, the supernatant was collected as cytosolic fraction. For nuclear fraction, 5 volumes of lysis buffer were added to the powder. After filtration and centrifugation at 13,000 × *g* for 10 min at 4 °C, the pellet was washed with NRBT buffer (20 mM Tris-HCl pH 7.4, 25% glycerol, 2.5 mM MgCl_2_, 0.2% Triton X-100, 5 mM DTT, 160 U/mL RNase inhibitor) and collected by centrifugation at 1,500 × *g* for 2 min at 4 °C. When the pellet was creamy white, 300 μL of Extraction Buffer II (250 mM sucrose, 10 mM Tris-HCl pH 8.0, 10 mM MgCl_2_, 1% Triton X-100, 5 mM β-mercaptoethanol, 1 × protease inhibitor, 350 U/mL RNase inhibitor) was added to resuspend the pellet. The suspension was added on the top of 300 μL of Extraction Buffer III (1.7 M sucrose, 10 mM Tris-HCl pH 8.0, 2 mM MgCl_2_, 0.15% Triton X-100, 5 mM β-mercaptoethanol, 1 × protease inhibitor, 350 U/mL RNase inhibitor) and the pure nuclei were collected by centrifugation at 13,000 × g for 10 min at 4 °C. As quality controls of the preparation of nuclear and cytosolic fractions, nuclear and cytosolic RNA markers U6 and tRNA were detected as described^63^.

### Preparation of polyadenylated and non-polyadenylated RNAs

Total RNA was extracted with TRIzol (Invitrogen) and treated with DNase I (Ambion). Polyadenylated [poly(A)+] RNAs were isolated from total RNA through two rounds of purification with oligo(dT) beads (QIAGEN). The poly(A)+ RNA-depleted fraction from the first round of purification was collected as crude non-polyadenylated [(poly(A)−] RNA sample, which was subjected to another two rounds of treatment with oligo(dT) beads. Ribosomal RNAs were removed by two rounds of treatment with the Ribo-minus kit (Invitrogen).

### Library preparation and sequencing

cDNA libraries for strand-specific sequencing were constructed by ligation- or dUTP-based methods^64^. A detailed protocol is available in Supplementary Methods.

### Reconstruction of *Arabidopsis* transcriptome

Clean reads (Phred quality score ≥ 20) were aligned to the *Arabidopsis* reference genome (TAIR10)^65^ by using TopHat version 2.0.10^66^. Parameters were set for strand-specific mapping and up to 5 different alignments were allowed for a given read. Annotations in TAIR10 served as an additional junction set to facilitate the alignment. Mapped reads from each RNA-seq dataset were assembled into transcripts in a reference annotation-based transcript assembly (RABT assembly) mode by Cufflinks version 1.3.0^67^. Putative transcripts were retrieved with the parameter ‘--min-frags-per-transfrag 1’. Finally, assembled transcripts from each dataset and the reference annotation were merged into a unified transcriptome using Cuffmerge utility version v1.0.0^42^.

### Identification of *Arabidopsis* lncRNAs

We developed a stringent selection pipeline to systemically identify *Arabidopsis* lncRNAs, on the basis of pipelines for animal lncRNA annotation^68,69^. This pipeline aimed at removing known non-lncRNA transcripts, unreliable lowly expressed transcripts, and transcripts with protein-coding potential. First, only transcripts with TAIR10 annotation [Cufflinks class codes ‘u’ (intergenic transcripts),’x’ (Exonic overlap with reference on the opposite strand),’i’ (transcripts entirely within intron) were retained. Second, transcripts of short length (length <150 nt) or low abundance (FPKM_max_ < 1, FPKM_max_ stands for the maximum expression level of a lncRNA from all samples) were removed. Third, transcripts with protein-coding potential were removed. Protein-coding potential was determined by using two programs: (1) transcripts were subjected to a BlastX search against all plant protein sequences in the Swiss-Prot database^70^ with a cutoff e-value < 10^-4^ and the transcripts with strong hits (alignment length ≥40 aa, percent identity ≥35% and coverage of the alignment region in either query or subject sequence ≥35%) to known proteins were considered to have protein-coding potential; For antisense transcripts, open reading frames were checked. (2) the CPC (Coding Potential Calculator) score^71^, a value to assess protein-coding potential of a transcript based on six biologically meaningful sequence features, was calculated for each transcript. When the CPC score is positive, we considered the transcript to have protein-coding potential. Transcripts that passed the three filtering steps were annotated as lncRNAs.

### Co-expression analysis

Pearson correlation coefficient was calculated between the expression levels of adjacent protein-coding genes and between the expression levels of lncRNAs and their closest protein-coding genes. LncRNA/protein-coding gene pairs with low abundance (FPKM_max_ < 1) were excluded from our analysis. LncRNA/protein-coding gene pairs with Pearson correlation coefficients greater than 0.6 were presented in the heat map.

### Quantitative RT-PCR

Total RNA was isolated using TRIzol reagent (Invitrogen) and treated with DNase I (Invitrogen) for 30 min at 37 °C to eliminate contaminated genomic DNA. cDNAs were generated using 2 μg of total RNA with random or gene-specific primers and M-MLV (Invitrogen). Quantitative RT-PCR was performed using SYBR Premix Ex Taq (Takara) as described. Each sample was analyzed in triplicate. The level of *GAPDH* mRNA was detected in parallel and used for normalization. Primer sequences are provided in Supplementary Data 9.

### sRNA sequencing and analysis

sRNAs of 18–30 nt were gel-purified on a 15% denaturing PAGE gel and subjected to library construction as described^47^. A detailed protocol is available upon request. The libraries were single-end sequenced on an Illumina HiSeq2000 platform. After removing adapters and low-quality reads, sRNA-seq reads were mapped to the *Arabidopsis* genome (TAIR10 version) with Bowtie^72^ allowing no mismatches, and the mapped reads were retained for further analyses. sRNA abundance was calculated as reads per million (RPM).

### ChIP and ChIP-qPCR analyses

ChIP was performed as described^73^ with some modifications. A detailed protocol is available in Supplementary Methods. qPCR was performed using SYBR Premix Ex Taq (Takara). Relative enrichment of H3K4me3, H3K27Ac, H3K27me3, H3K36me3 and WDR5a in each DNA region was normalized to input DNA. Primer sequences are provided in Supplementary Data 9.

### RNA IP

RNA IP (RIP) was performed as described^74^. Briefly, plants were harvested and crosslinked by using 1% formaldehyde for 20 min. RNA-protein complexes were immunoprecipitated by incubating with anti-FLAG M2 Magnetic Beads (M8823, Sigma-Aldrich) and rabbit polyclonal anti-H3 (ab1791, Abcam, 1:200) at 4 °C for 6 h. Then, the crosslinking was reversed and RNA was purified by TRIzol.

### Nuclear run-on assay

Nuclear run-on assay was performed as described^75^ except that nuclei were isolated from 10-day-old seedlings as described^62^. A detailed protocol is available in Supplementary Methods.

### ChIRP and ChIRP-qPCR analyses

ChIRP was performed as previously with some modifications^76^. Antisense DNA probes which were separated into two groups (even and odd) were designed against the full-length *MAS* sequence and biotinylated at the 3’ end (Invitrogen). A set of probes against *lacZ* RNA was used as negative control.

Col-0 seedlings (1 g) were crosslinked in 1% (vol/vol) formaldehyde (Sigma-Aldrich) at room temperature for 20 min in a vacuum. Crosslinking was quenched with 0.125 M glycine for 5 min. Nuclei were isolated as described in the NRO assay and were sonicated. Chromatin was diluted in 2 volumes of hybridization buffer (750 mM NaCl, 1% SDS, 50 mM Tris-HCl pH 7.0, 1 mM EDTA, 15% formamide, 0.1 mM PMSF, 1 × protease inhibitor, and 350 U/mL RNase inhibitor) and was mixed gently. After preclearance with Streptavidin Sepharose beads (GE Healthcare), 100 pmol of probes were added and mixed by end-to-end rotation at 37 °C for 4 h. Washed Streptavidin Sepharose beads (30 μL) were added, and the reaction was performed at 37 °C for 30 min with rotation. Then beads were washed two times with high-salt wash buffer (2 × SSC, 0.5% SDS, 1 mM DTT, and 1 mM PMSF) and two times with low-salt wash buffer (0.1 × SSC, 0.5% SDS, 1 mM DTT, and 1 mM PMSF) for 5 min each time at room temperature. DNA and RNA were purified and analyzed by qPCR. Probes and primer sequences are provided in Supplementary Data 9.

### RNA decay assay

RNA decay assay was performed as described^77^ with some modifications. Two-week-old seedlings of Col-0, *amiR-MAS-1*, and *amiR-MAS-2* were grown in 4 °C growth chamber for 20 d. After cold treatment, plants were transferred into 1/2 MS medium with 100 μg/mL actinomycin D (Sigma-Aldrich). Materials were harvested after 2, 4, 6, 8 h. Total RNA was extracted by TRIzol reagent and used for RT-qPCR assays.

### 5’ and 3’ RACE

Poly(A)+ RNAs were isolated from 100 ug total RNAs using oligo(dT) Dynabeads (Thermo Fisher). The 5’ and 3’ RACE experiments were preformed according to the manuals of GeneRacer (Invitrogen). For 3’ RACE, poly(A)+ RNAs were reversely transcribed with GeneRacer oligo (dT) primers and then amplified with GeneRacer 3’Primer/Nest primer and MAS-3’ RACE-GSP1/2/3. For 5’ RACE, poly(A)+ RNAs were reversely transcribed with MAS-5’ RACE-GSP1. After degradation of RNAs, the cDNA was tailed by dCTP and the second strand cDNA was generated using the Abridged Anchor Primer (AAP). Final amplification was performed with the Abridged Universal Anchor Primer (AUAP) and MAS-5’ RACE-GSP2/3. Primer sequences are provided in Supplementary Data 9.

## Electronic supplementary material

Supplementary Informaton

Peer Review

Description of Additional Supplementary Files

Source Data

Supplementary Data 1

Supplementary Data 2

Supplementary Data 3

Supplementary Data 4

Supplementary Data 5

Supplementary Data 6

Supplementary Data 7

Supplementary Data 8

Supplementary Data 9

Reporting Summary

## Data Availability

RNA-Seq and sRNA-seq datasets generated in this study can be found in the NCBI Gene Expression Omnibus under accession number GSE42695 and GSE120709. A reporting summary for this Article is available as a Supplementary Information file. The source data underlying Figs. 2–6 and Supplementary Figs. 2-3, 5-6 and 8-10 are provided as a Source Data file. All other data that support the findings of this study are available from the corresponding author upon request.
