# Supplementary material for: Global identification of Arabidopsis lncRNAs reveals the regulation of MAF4 by a natural antisense RNA
Source: Nat Commun. 2018 Nov 29;9:5056. doi: 10.1038/s41467-018-07500-7 (PMC6265284; doi:10.1038/s41467-018-07500-7)
Supplement: Supplementary file 1 — Supplementary Informaton [file 41467_2018_7500_MOESM1_ESM.docx]

**Supplementary Information**

**Supplementary Figures**

**
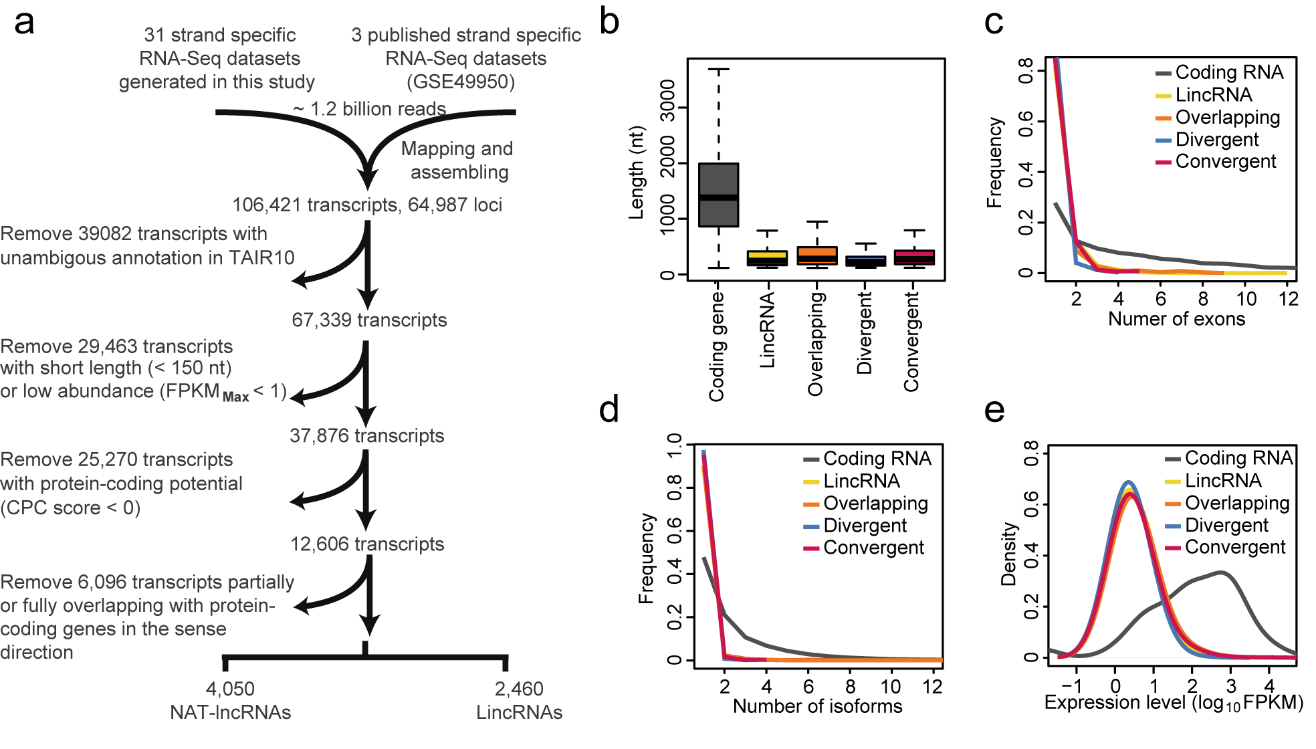
**

**Supplementary Figure 1. Identification and characteristics of *Arabidopsis* lncRNAs.**

**a**, A pipeline for systematic identification of lncRNAs in *Arabidopsis*. **b**, A boxplot showing the length distribution of in different lncRNA groups. **c**, A frequency plot showing the distribution of exon numbers in different lncRNA groups. **d**, A frequency plot showing the distribution of isoform numbers in different lncRNA groups. **e**, Cumulative distribution of expression levels of different lncRNA groups. The x axis represents the maximal expression (log10-normalized FPKM counts) of lncRNAs across the samples in this study.

**
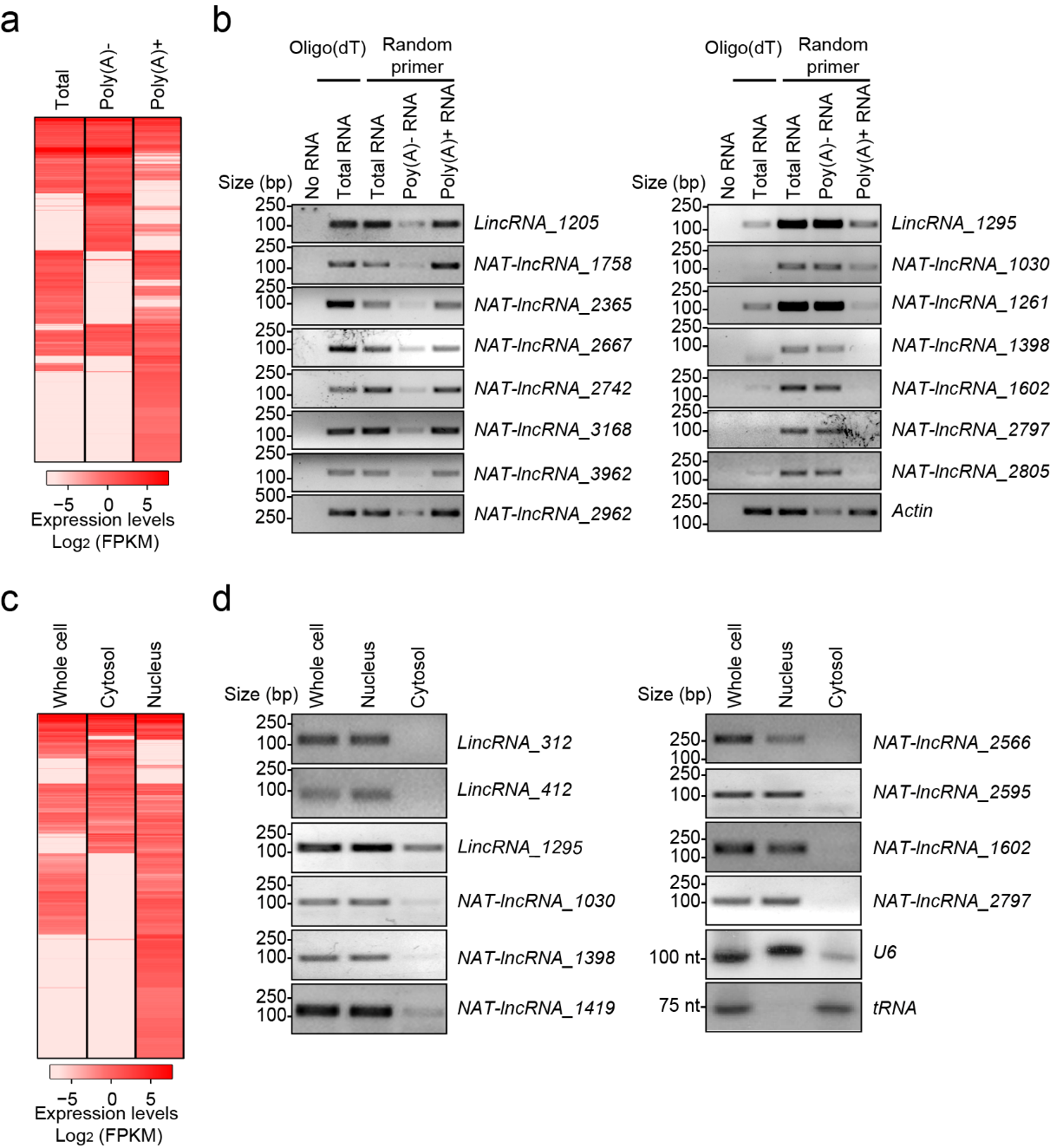
**

**Supplementary Figure 2. Features of *Arabidopsis* lncRNAs.**

**a**, A heat map showing the relative abundances of lncRNAs in total, poly(A)- RNA and poly(A)+ RNA samples. Color intensity represents log2-normalized FPKM counts. Rows are ordered based on a hierarchical clustering of lncRNAs **b**, Validation of poly(A)+ and poly(A)- lncRNAs by RT-PCR. Total RNA, poly(A)-enriched [poly(A)+] and poly(A)-depleted [poly(A)-] RNA fractions were used for RT-PCR analysis. **c**, Heat map showing relative abundance of lncRNAs in total, cytosolic and nuclear RNA samples. Color intensity represents log2-normalized FPKM counts. Rows are ordered based on a hierarchical clustering of lncRNAs. **d**, Validation of nuclear enrichment of lncRNAs by RT-PCR analysis. U6 and tRNA were used as nuclear and cytosolic RNA markers, respectively. Source data are provided as a Source Data file.

**
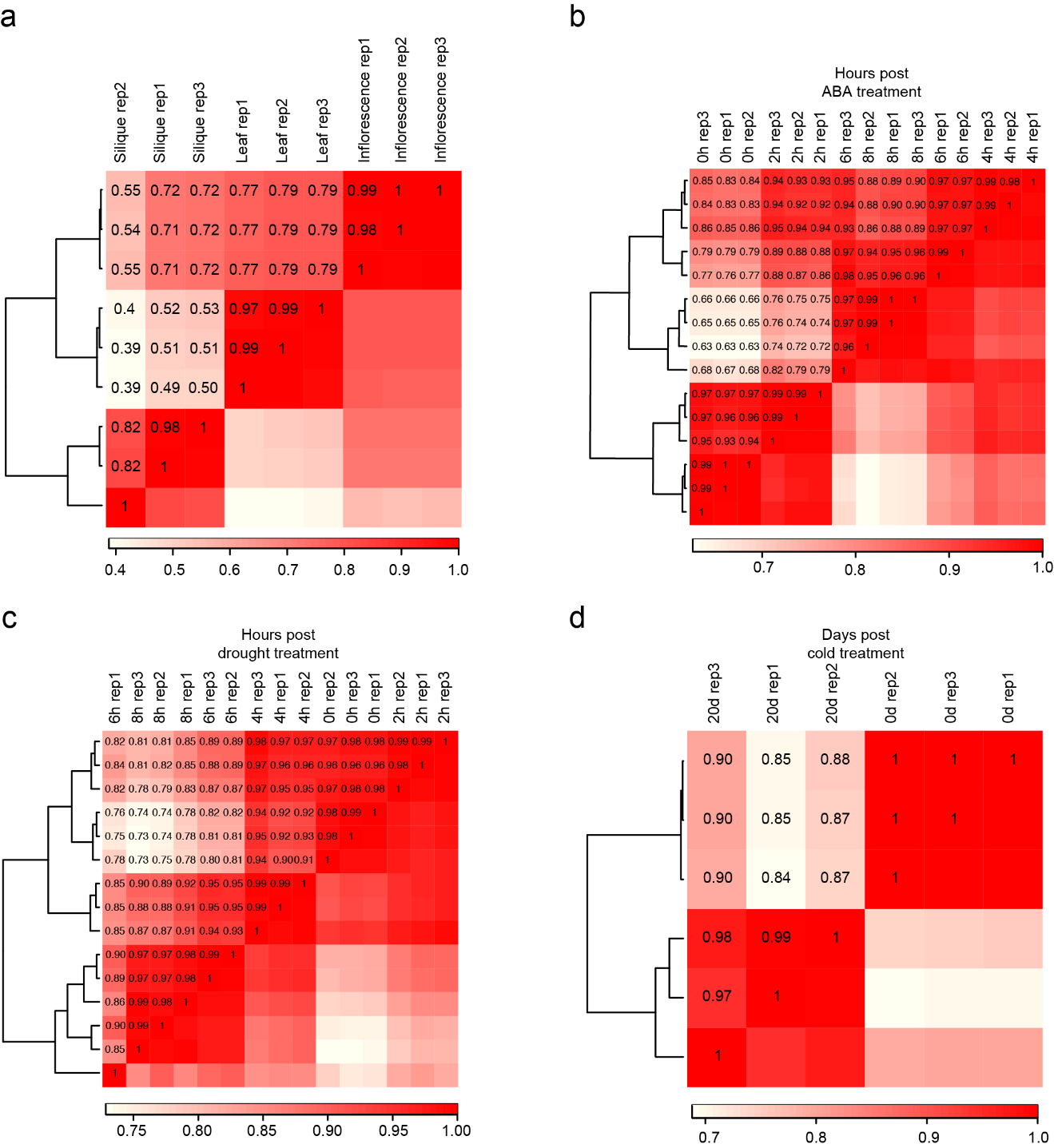
**

**Supplementary Figure 3. The Pearson correlation coefficient between different biological replicates of RNA-seq data.**

Heatmap representation of the Pearson correlation coefficient between different biological replicates of RNA-seq data. RNA-seq was performed using materials from three tissues (a), and materials treated with ABA (b), drought (c) and cold (d). Hierarchical clustering analyses reveal high correlations between three biological replicates of RNA-seq data. The color bar shows the strength of the correlation and the deeper the color, the higher the correlation. Source data are provided as a Source Data file.

**
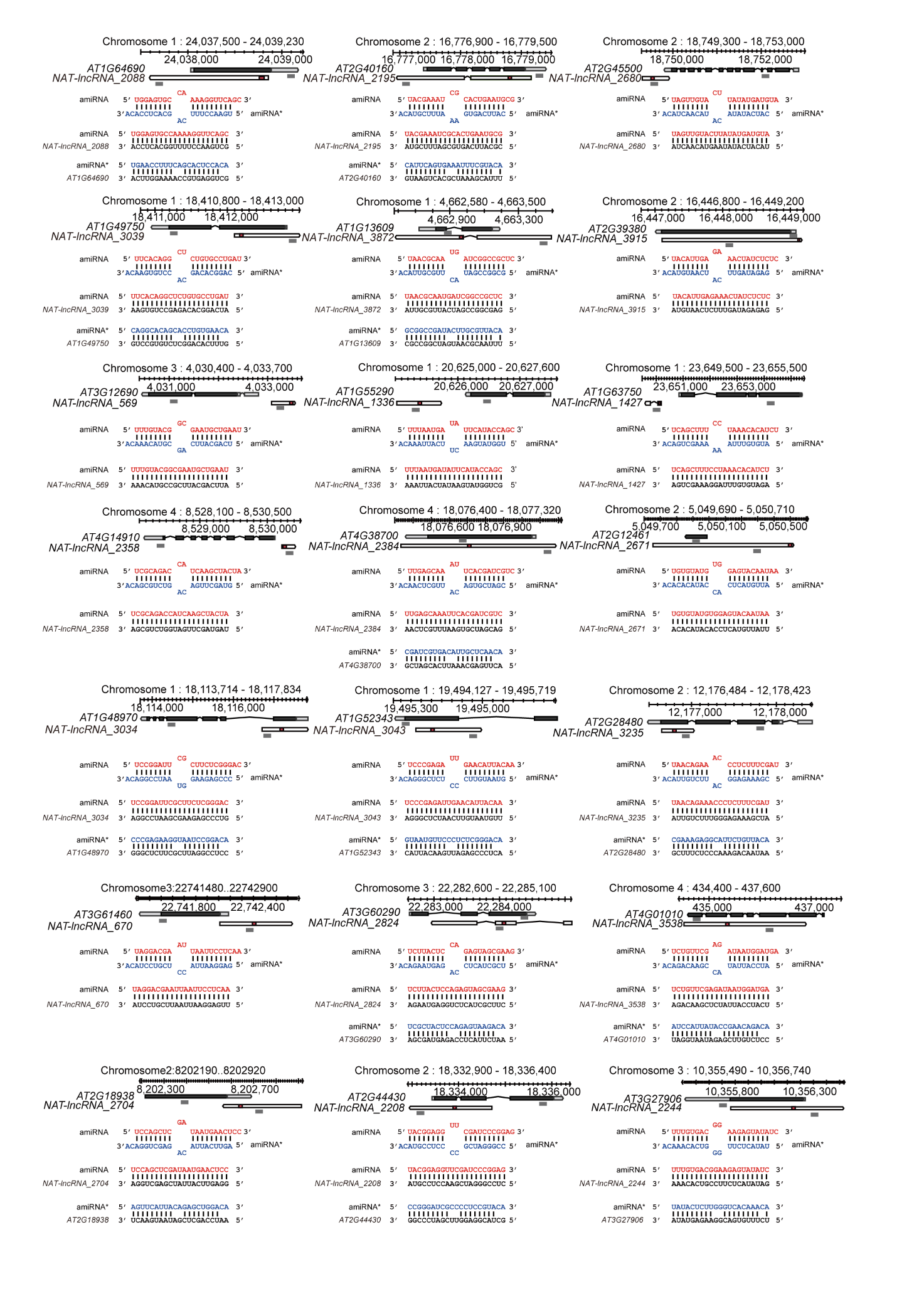
**

**Supplementary Figure 4. Sequences and structures of amiRNA duplexes and the target sites of amiRNAs and amiRNA*s.**

Upper panels, genome browser views of NAT-lncRNAs and their cognate sense genes. Lower panels, sequences of amiRNA duplexes, amiRNA target sites on NAT-lncRNAs and potential amiRNA* target sites on cognate sense genes. Grey bars depict the relative positions of primers used for RT-qPCR analyses in Fig.3 and Supplementary Fig. 5,6.

**
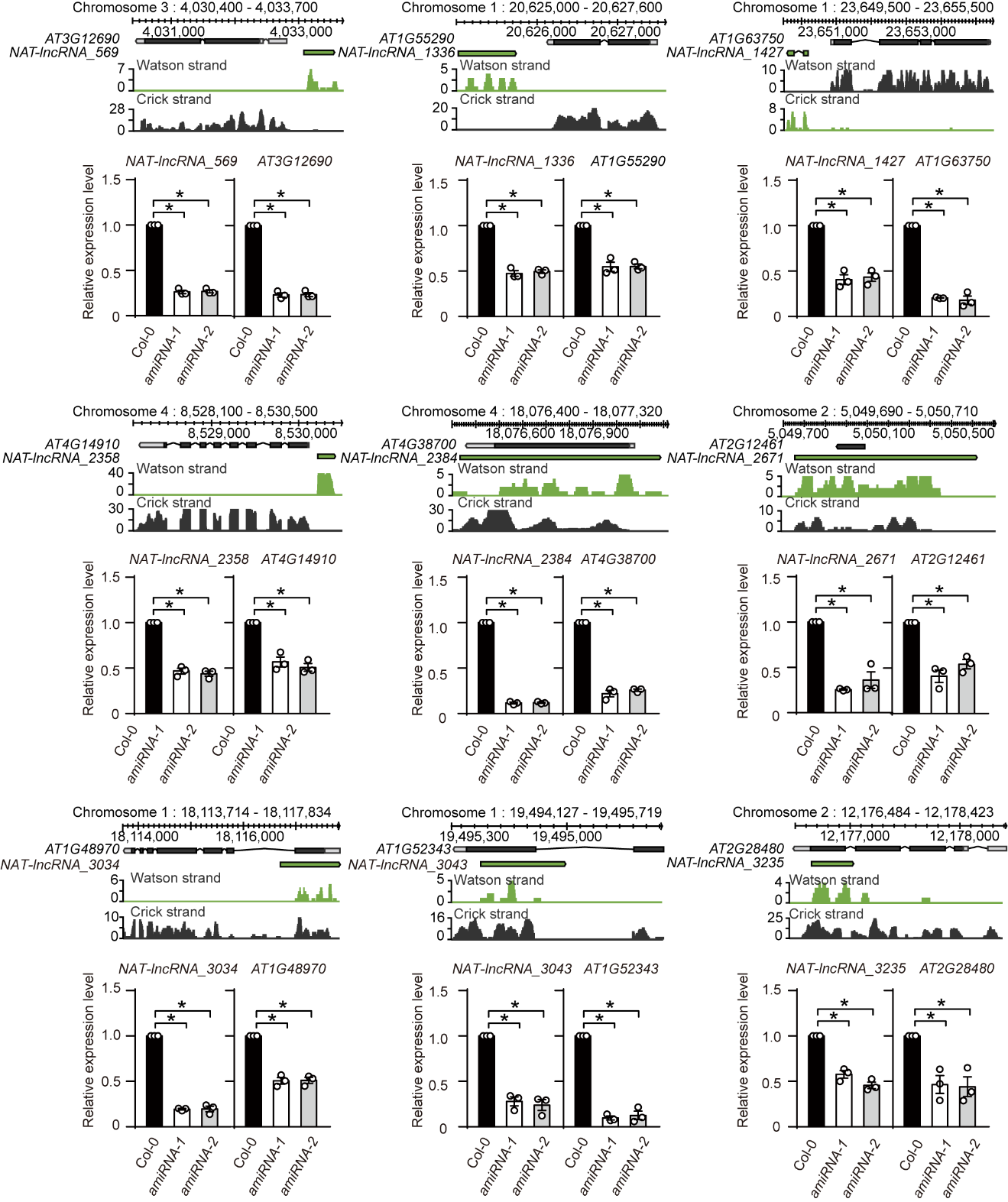
**

**Supplementary Figure 5. NAT-lncRNAs regulate the expression of cognate sense genes.**

Detection of NATs-lncRNAs and their cognate sense genes by RT-qPCR in Col-0 and the indicated *amiRNA* knockdown lines. Error bars represent s.e.m (n=3), asterisks indicate a significant difference (*t* test, *P* value < 0.05). Source data are provided as a Source Data file. Shown above the RT-qPCR results are genome browser views of RNA-seq signals at NAT-lncRNAs and cognate sense genes in Col-0, with normalized read counts per million along the y axis.

**
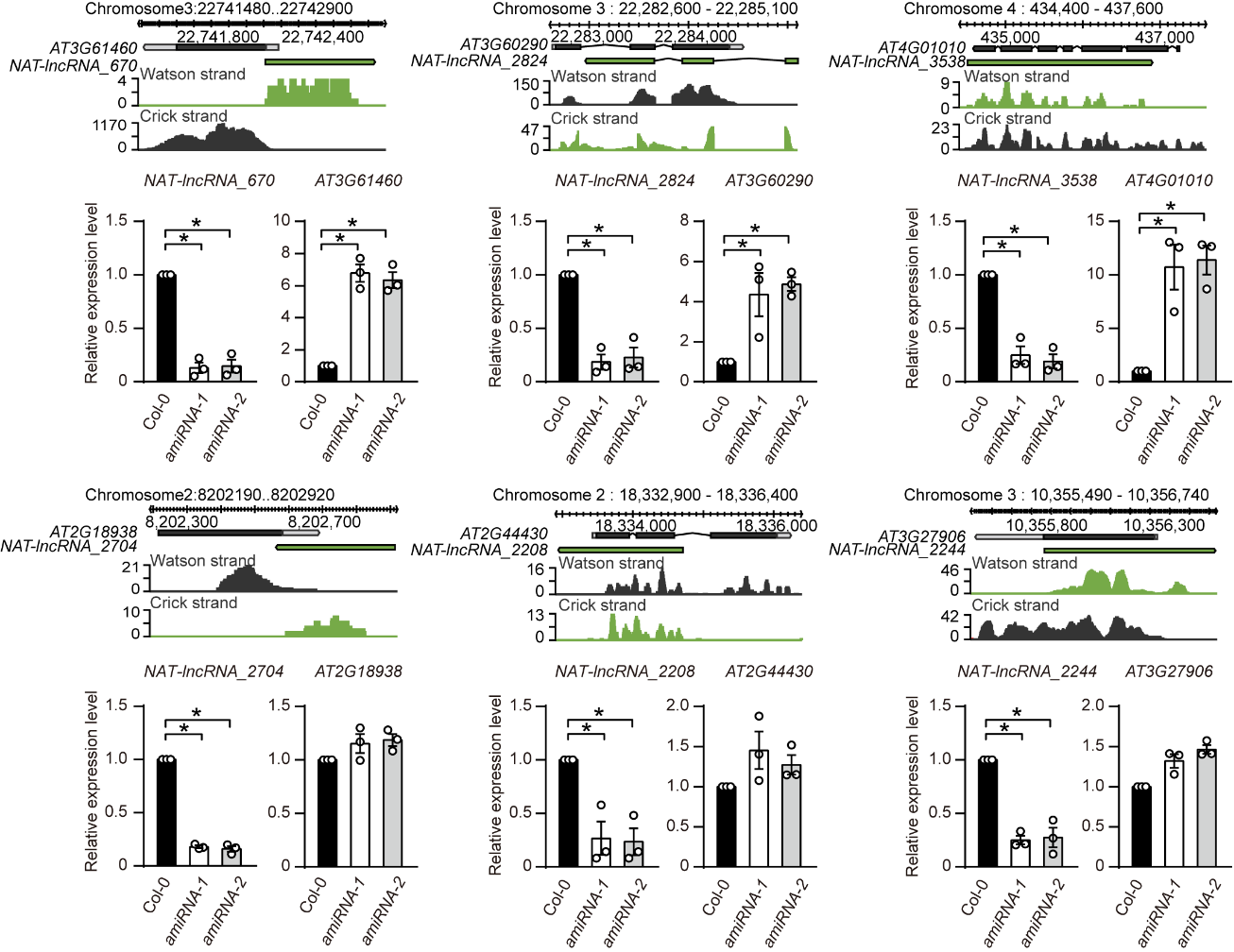
**

**Supplementary Figure 6. NAT-lncRNAs regulate the expression of cognate sense genes.**

Detection of NATs-lncRNAs and their cognate sense genes by RT-qPCR in Col-0 and the indicated *amiRNA* knockdown lines. Error bars represent s.e.m (n=3), asterisks indicate a significant difference (*t* test, *P* value < 0.05). Source data are provided as a Source Data file. Shown above the RT-qPCR results are genome browser views of RNA-seq signals at NAT-lncRNAs and cognate sense genes in Col-0, with normalized read counts per million along the y axis.

**
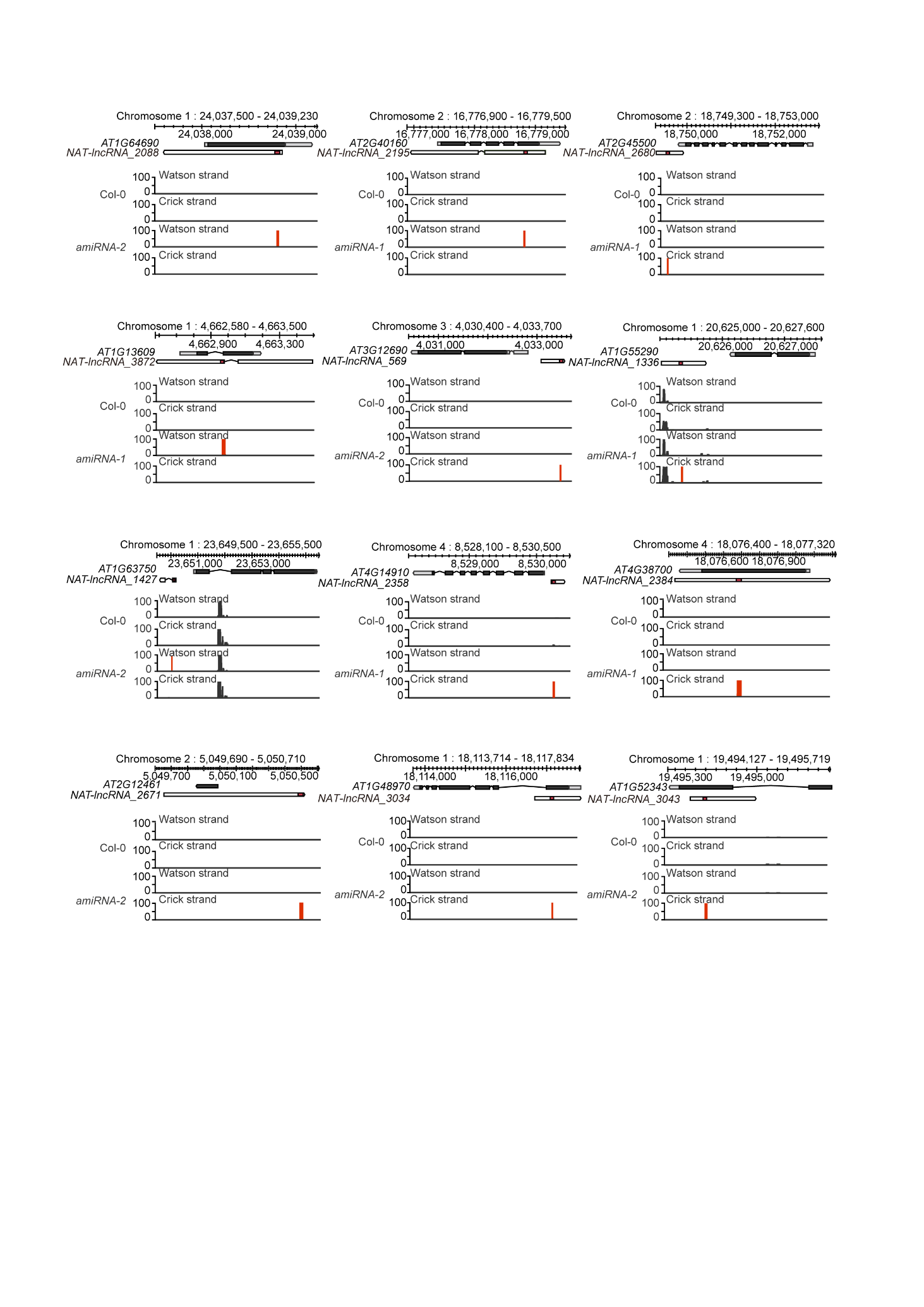
**

**Supplementary Figure 7. amiRNAs do not trigger the production of secondary siRNAs.**

Genome browser views of small RNA-seq signals at twelve randomly chosen NAT-lncRNAs and cognate sense genes in Col-0 and the indicated *amiRNA* knockdown lines, with normalized read counts per million along the y axis. amiRNA signals and target sites on NAT-lncRNAs are shown in red.

**
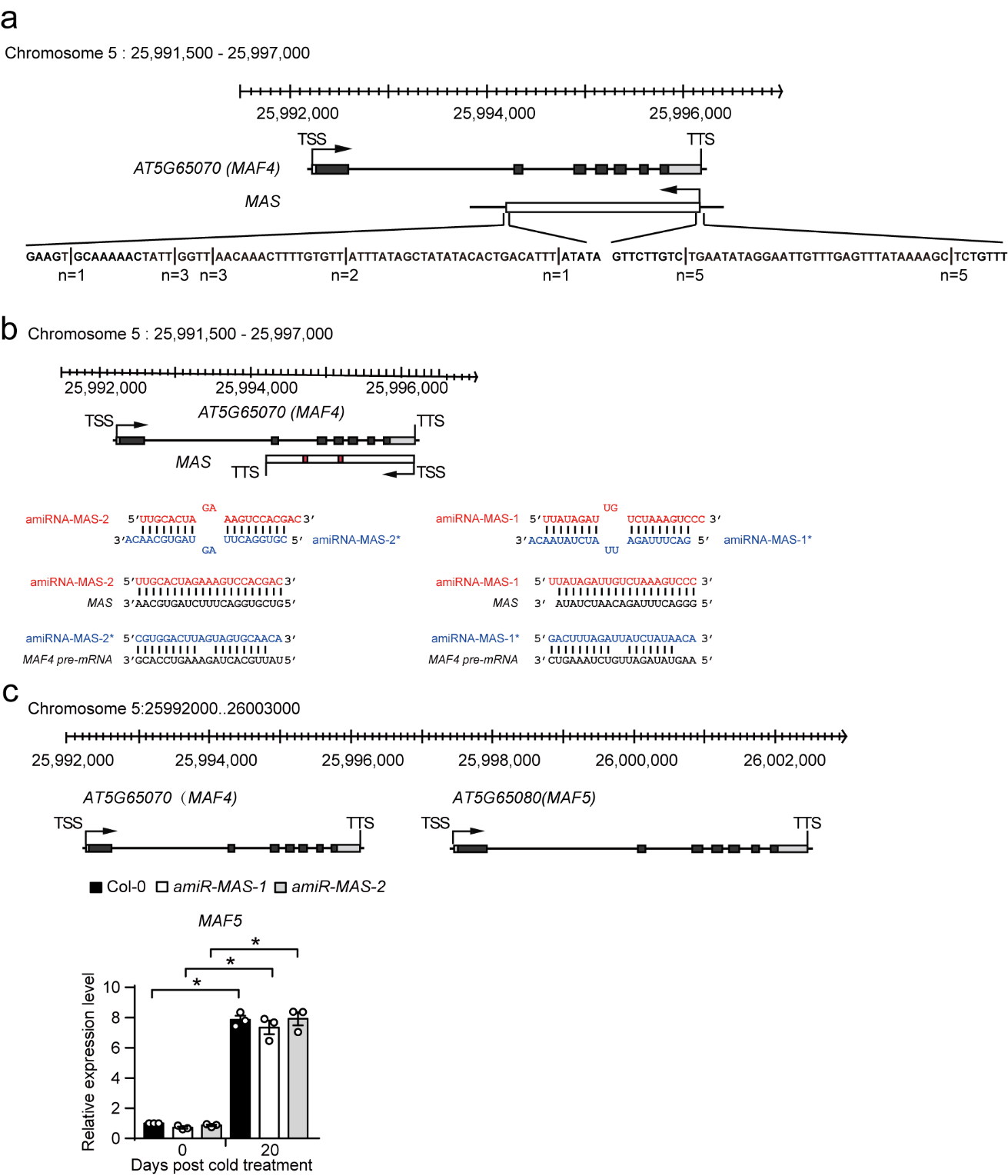
**

**Supplementary Figure 8. Identification of the 5’ and 3’ ends of *MAS* transcript, knockdown of *MAS* by amiRNAs and the effect of *MAS* knockdown on *MAF5* expression.**

**a,** Transcriptional start and stop sites of *MAS* were determined by 5’ and 3’ RACE. Ten independent sub-clones of 5’ or 3’ RACE products were sequenced. **b,** Upper panel, genome browser view of *MAF4* and *MAS*. Lower panel, sequences of two amiRNA (amiR-MAS-1 and amiR-MAS-2) duplexes for *MAS* knockdown, amiRNA target sites on *MAS* and potential amiRNA*** target sites on *MAF4* mRNA. **c,** Detection of *MAF5* expression by RT-qPCR in Col-0 and two *MAS* *amiRNA* knockdown lines *amiR-MAS-1/2* before (0 d) and after 20 d of cold exposure. Error bars represent s.e.m (n=3), asterisks indicate a significant difference between the indicated groups (*t* test, *P* value < 0.05). Source data are provided as a Source Data file.

**
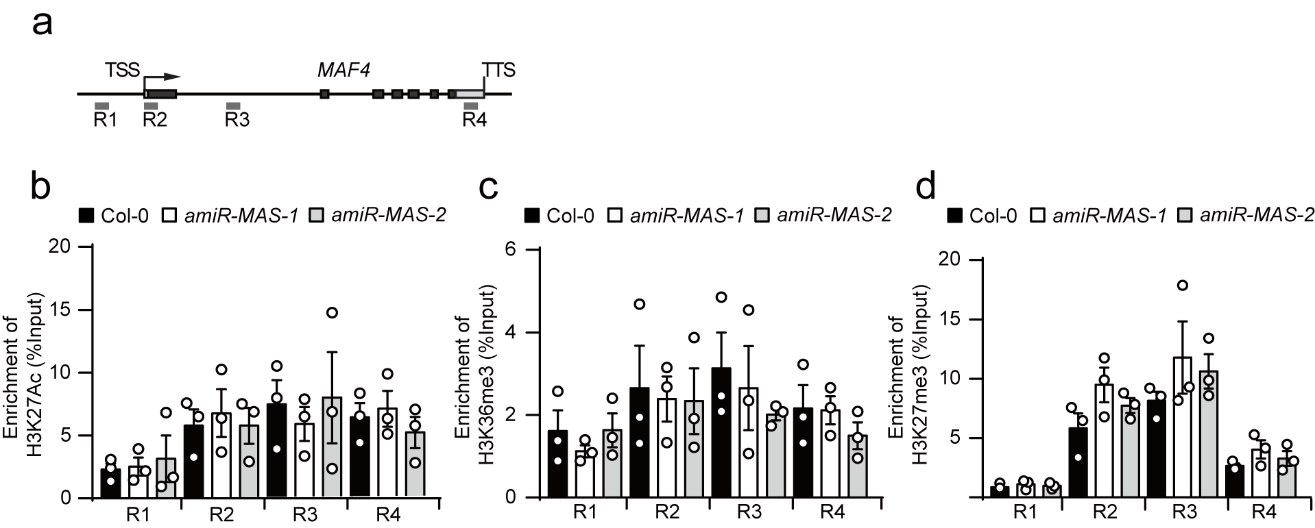
**

**Supplementary Figure 9. *MAS* does not regulate H3K27Ac, H3K36me3 and H3K27me3 deposition at *MAF4*.**

**a**, schematic representation of *MAF4* locus. The positions of primers (R1 to R4) used for ChIP-qPCR are indicated. **b**-**d**, Detection of H3K27Ac, H3K36me3 and H3K27me3 levels in Col-0 and *amiR-MAS-1/2* lines after 20 d of cold exposure by ChIP-qPCR. Error bars represent s.e.m (n=3). Source data are provided as a Source Data file.


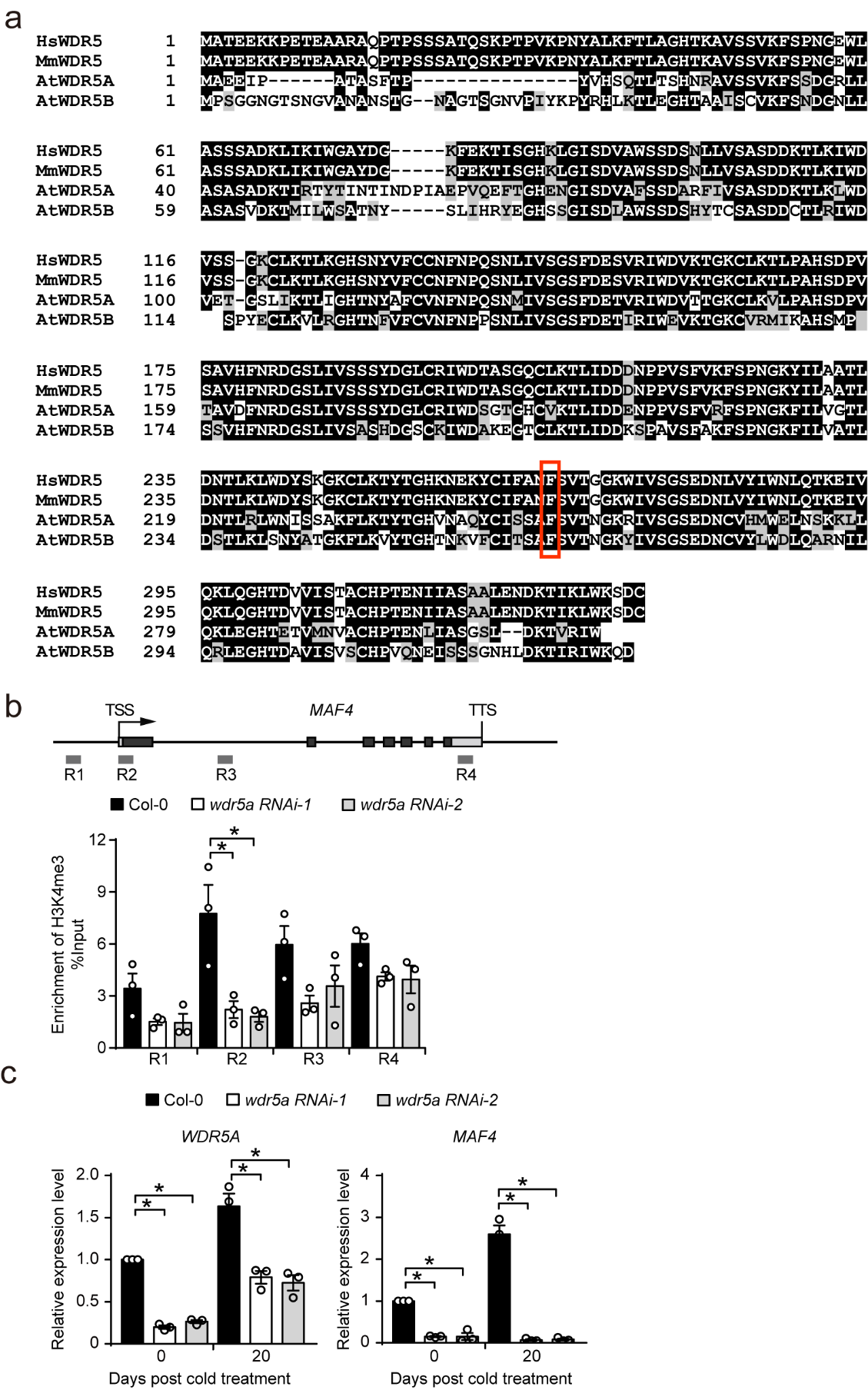


**Supplementary Figure 10. WDR5a is involved in the regulation of *MAF4* expression.**

**a**, Multiple sequence alignment of *Arabidopsis* WDR5a (AtWDR5a, At3g49660) and WDR5b (AtWDR5B, At4g02730) with *Homo sapiens* WDR5 (HsWDR5) and *Mus musculus* WDR5 (MmWDR5). Identical residues are shaded by black, and similar residues are shaded by gray. A conserved phenylalanine residue required for lncRNA binding is highlighted by a red frame. **b**, Upper panel, schematic representation of the *MAF4* locus. The positions of primers (R1 to R4) used for ChIP-qPCR are indicated. Lower panel, Detection of H3K4me3 levels in Col-0 and *wdr5a RNAi*-1/2 lines after 20 d of cold exposure by ChIP-qPCR. **c**, Detection of *WDR5a* and *MAF4* in Col-0 and *wdr5a RNAi*-1/2 lines before (0 d) and after 20 d of cold exposure. In **b**,**c**, error bars represent s.e.m (n=3), asterisks indicate a significant difference between the indicated groups (*t* test, *P* value < 0.05). Source data are provided as a Source Data file.

**Supplementary Methods**

**Plant materials and generation of transgenic lines**

The *maf4-1* mutant (Salk_028506C) was obtained from ABRC. The *wdr5a* RNAi-1/2 lines were described^1^.

To generate transgenic lines expressing FLAG-tagged WDR5a, genomic sequence of *WDR5a* fused with an N-terminal 3×FLAG tag was cloned into the pENTR-D TOPO (Invitrogen). WDR5A^F250A^ was generated by site-directed mutagenesis. *FLAG-WDR5a* and *FLAG-WDR5a^F250A^* were recombined into pMDC32 by LR reactions. amiRNAs constructs targeting NAT-lncRNAs and *MAF4* were constructed, using pre-miR159 as backbone as described^2^. The genomic sequence containing *MAF4* with its putative promoter and *MAS* was reversely inserted into the pER8 vector^3^ by using In-Fusion cloning (Clontech) to generate an inducible expression construct for *MAS*. The genomic sequence of *MAS* was put downstream of a 35S promoter in pMDC32 to obtain the *MAS* overexpression construct. The primers used for cloning are listed in Supplementary Data ^9^. All constructs were introduced into *Agrobacterium* strain GV3101 for plant transformation. Transgenic plants were constructed using a floral dip method ^4^. Positive transformants were selected by antibiotic markers and confirmed by RT-qPCR, Northern blot or Western blot.

**Library preparation and sequencing**

RNAs were first fragmented by partial alkaline hydrolysis at 95°C. The resulting fragmented RNAs were separated on 12% Urea-PAGE and RNAs of 60 nt in length were recovered. The 3’ adaptor (5’-pAGA UCG GAA GAG CGG UUC AGC-3’) and the 5’ adaptor (5’-ACG ACG CUC UUC CGA UCU-3’) were ligated to the recovered 60 nt RNAs. After reverse transcription by the RT-primer (5’-GCT GAA CCG CTC TTC CGA TCT-3’), cDNAs were amplified with primers (primer-F 5’-AAT GAT ACG GCG ACC ACC GAG ATC TAC ACT CTT TCC CTA CAC GAC GCT CTT CCG ATC T-3’, primer-R 5’-CAA GCA GAA GAC GGC ATA CGA GAT CGG TCT CGG CAT TCC TGC TGA ACC GCT CTT CCG ATCT-3’) so that the products for sequencing were compatible with the Illumina sequencing platform. The quality of libraries was checked by BioAnalyzer 2100 (Agilent) and the libraries were sequenced on the Illumina Genome Analyzer IIx platform. cDNA libraries for ABA, drought, and cold treated samples were constructed by a dUTP based method with a commercial kit (NEXTflex^TM^ Rapid Directional RNA-Seq Kit, Bioo). The quality of each library was examined by BioAnalyzer 2100 (Agilent) and sequencing was performed on Illumina sequencing platform.

**ChIP**

Two-week-old seedlings subjected to 20 d cold treatment (about 2 g) were ground into fine powder in liquid nitrogen and suspended in 10 mL of nuclear isolation buffer (20 mM Tris-HCl pH 7.5, 20 mM KCl, 2 mM EDTA, 2.5 mM MgCl_2_，25% glycerol, 250 mM sucrose and 1x protease inhibitor). For crosslinking, formaldehyde was added to reach the final concentration 1%. The samples were incubated at 4°C for 20 min with rotation and the reactions were stopped by the addition of 2 M Glycine (final concentration is 0.125 M). The extracts were then filtered through two layers of Miracloth and centrifuged at 1,500 g for 10 min at 4°C. The nuclear pellets were washed with 10 mL of extraction buffer (20 mM Tris-HCl pH 7.5, 2.5 mM MgCl_2_, 25% glycerol, 0.2% Triton X-100 and 1× protease inhibitor) and centrifuged at 1,500 g for 10 min at 4°C for several times. The pellets were then resuspended in 300 μL of nuclear lysis buffer (50 mM Tris-HCl pH 8.0, 10 mM EDTA, 1% SDS, 1× protease inhibitor and 0.1 mM PMSF). DNA was fragmented by sonication for 20 min. Then, the nuclear extract was diluted with ChIP dilution buffer (1.1 % Triton X-100, 1.2 mM EDTA, 16.7 mM Tris-HCl pH 8.0, 167 mM NaCl, 0.1 mM PMSF and 1× protease inhibitor) to a final volume of 3 mL. After centrifugation at 12,000 rpm at 4°C for 10 min, the supernatant was incubated with one of the following antibodies: rabbit polyclonal anti-WDR5 (ab75439, Abcam, 1:150), rabbit polyclonal anti-H3K4me3 (04-745, Millipore, 1:250) , rabbit polyclonal anti-H3K27ac (ab4729, Abcam, 1:250), rabbit polyclonal anti-H3K36me3 (ab9050, Abcam, 1:250) and rabbit polyclonal anti-H3K27me3 (07-449, Millipore, 1:250) at 4°C for 6 h with rotation. The antibody-protein complexes were isolated by protein A magnetic beads (16-661, Millipore) or anti-FLAG M2 Magnetic Beads (M8823, Sigma). Beads were washed twice with each of the following solutions for 5 min at 4°C: high salt buffer (500 mM NaCl, 0.1% SDS, 1% Triton X-100, 2 mM EDTA and 20 mM Tris-HCl pH 8.0); low salt buffer (150 mM NaCl, 0.1% SDS, 1% Triton X-100, 2 mM EDTA and 20 mM Tris-HCl pH 8.0); LiCl-containing buffer (0.25 mM LiCl, 1% NP-40, 1% sodium doxycholate, 1 mM EDTA, and 10 mM Tris-HCl pH 8.0), and TE Buffer (10 mM Tris-HCl pH 8.0, and 1 mM EDTA). Then, IPed proteins were eluted with 200 µL of elution buffer (1% SDS and 0.1 M NaHCO_3_). The crosslinking was reversed in 0.2 M NaCl at 65°C overnight and then digested with proteinase K at 45°C for 1 h. Subsequently, the DNA was purified by phenol/chlorophorm extraction and precipitated by ethanol.

**Nuclear run-on assay**

About 1 g of seedlings was ground into fine powder in liquid nitrogen and suspended in 10 mL of nuclear isolation buffer (20 mM Tris-HCl pH 7.5, 20 mM KCl, 2 mM EDTA pH 8.0, 2.5 mM MgCl_2_, 25% glycerol, 250 mM sucrose and 1× protease inhibitor). The extracts were then filtered through two layers of Miracloth and centrifuged at 1,500 g for 10 min at 4°C. The nuclear pellets were washed with 10 mL of extraction buffer (20 mM Tris-HCl pH 7.5, 2.5 mM MgCl_2_, 25% glycerol, 0.2% Triton X-100 and 1× protease inhibitor) and centrifuged at 1,500 g for 10 min at 4°C. The pellets were then resuspended in 50 μL nuclei storage buffer (50 mM Tris-HCl pH 7.8, 10 mM 2-mercaptoethanol, 20% glycerol, 5 mM MgCl_2_, 0.44 M sucrose). The run-on assay was performed in 1× transcription assay buffer (50 mM Tris-HCl pH 7.5, 5 mM MgCl_2_, 150 mM KCl, 0.1% sarkosyl, 2 U/mL RNase inhibitor, 10 mM DTT, 10 mM ATP, 10 mM CTP, 10 mM GTP, 10 mM BrUTP) at 30°C for 30 min. The reaction was stopped by adding 600 μL of TRIzol reagent, and RNA was extracted followed by the DNase I treatment to remove genomic DNA. The purified RNA was incubated with 2 μg mouse monoclonal anti-BrdU antibody (ab1893, Abcam, 1:250) at 4°C for 2 h and then immune-precipitated with Dynabeads Protein G (Invitrogen) pre-coated with yeast tRNA (Sigma) for 1 h. Co-precipitated RNA was extracted by TRIzol and used for RT-qPCR.

**Supplementary References**

1. Jiang, D., Gu, X. & He, Y. Establishment of the winter-annual growth habit via FRIGIDA-mediated histone methylation at FLOWERING LOCUS C in Arabidopsis. *Plant Cell* **21**, 1733-46 (2009).

2. Niu, Q.W. *et al.* Expression of artificial microRNAs in transgenic Arabidopsis thaliana confers virus resistance. *Nat Biotechnol* **24**, 1420-8 (2006).

3. Zuo, J., Niu, Q.W. & Chua, N.H. Technical advance: An estrogen receptor-based transactivator XVE mediates highly inducible gene expression in transgenic plants. *Plant J* **24**, 265-73 (2000).

4. Clough, S.J. & Bent, A.F. Floral dip: a simplified method for Agrobacterium-mediated transformation of Arabidopsis thaliana. *Plant J* **16**, 735-43 (1998).
