# Supplementary material for: Global identification of Arabidopsis lncRNAs reveals the regulation of MAF4 by a natural antisense RNA
Source: Nat Commun. 2018 Nov 29;9:5056. doi: 10.1038/s41467-018-07500-7 (PMC6265284; doi:10.1038/s41467-018-07500-7)
Supplement: Supplementary file 3 — Description of Additional Supplementary Files [file 41467_2018_7500_MOESM3_ESM.docx]

**Title:** Supplementary Data 1

**Description:** Summary of RNA-seq data generated in this study.

**Title:** Supplementary Data 2

**Description:** List and expression profile of lncRNAs.

**Title:** Supplementary Data 3

**Description:** List, expression levels and statistical analysis of poly(A)+/poly(A)- lncRNAs.

**Title:** Supplementary Data 4

**Description:** List, expression levels and statistical analysis of nuclear/cytosolic enriched lncRNAs.

**Title:** Supplementary Data 5

**Description:** List, expression levels and statistical analysis of tissue-specific lncRNAs.

**Title:** Supplementary Data 6

**Description:** List, expression levels and statistical analysis of lncRNAs differentially expressed response to ABA treatment.

**Title:** Supplementary Data 7

**Description:** List, expression levels and statistical analysis of lncRNAs differentially expressed response to drought treatment.

**Title:** Supplementary Data 8

**Description:** List, expression levels and statistical analysis of lncRNAs differentially expressed response to cold treatment.

**Title:** Supplementary Data 9

**Description:** Primers used in this study.

**Title:** Source data

**Description:** Statistical analysis and uncropped gels underlying Figures 2-6 and Supplementary Figures 2-3, 5-6 and 8-10.
