# Supplementary material for: Global identification of Arabidopsis lncRNAs reveals the regulation of MAF4 by a natural antisense RNA
Source: Nat Commun. 2018 Nov 29;9:5056. doi: 10.1038/s41467-018-07500-7 (PMC6265284; doi:10.1038/s41467-018-07500-7)
Supplement: Supplementary file 14 — Reporting Summary [file 41467_2018_7500_MOESM14_ESM.pdf]

nature research

Corresponding author(s): Yijun Qi

Reporting Summary

Nature Research wishes to improve the reproducibility of the work that we publish. This form provides structure for consistency and transparency in reporting. For further information on Nature Research policies, see Authors & Referees and the Editorial Policy Checklist.

Statistical parameters

When statistical analyses are reported, confirm that the following items are present in the relevant location (e.g. figure legend, table legend, main text, or Methods section).

n/a	Confirmed	
☐	☒	The exact sample size (*n*) for each experimental group/condition, given as a discrete number and unit of measurement
☐	☒	An indication of whether measurements were taken from distinct samples or whether the same sample was measured repeatedly
☐	☒	The statistical test(s) used AND whether they are one- or two-sided<br>*Only common tests should be described solely by name; describe more complex techniques in the Methods section.*
☐	☒	A description of all covariates tested
☐	☒	A description of any assumptions or corrections, such as tests of normality and adjustment for multiple comparisons
☐	☒	A full description of the statistics including central tendency (e.g. means) or other basic estimates (e.g. regression coefficient) AND variation (e.g. standard deviation) or associated estimates of uncertainty (e.g. confidence intervals)
☐	☒	For null hypothesis testing, the test statistic (e.g. *F*, *t*, *r*) with confidence intervals, effect sizes, degrees of freedom and *P* value noted<br>*Give P values as exact values whenever suitable.*
☒	☐	For Bayesian analysis, information on the choice of priors and Markov chain Monte Carlo settings
☒	☐	For hierarchical and complex designs, identification of the appropriate level for tests and full reporting of outcomes
☐	☒	Estimates of effect sizes (e.g. Cohen's *d*, Pearson's *r*), indicating how they were calculated
☐	☒	Clearly defined error bars<br>*State explicitly what error bars represent (e.g. SD, SE, CI)*

Our web collection on statistics for biologists may be useful.

Software and code

Policy information about availability of computer code

Data collection	Trimmomatic-0.30, TopHat v2.0.10, cufflinks v2.2.1, R version 3.1.0, Bedtools Version 2.19.1, perl, v5.10.1, and edgeR.
Data analysis	Trimmomatic-0.30 was used for NGS raw reads processing, TopHat v2.0.10 was used for ssRNA-seq short reads mapping, cufflinks v2.2.1 was used for novel lncRNAs annotation, R version 3.1.0 was used for statistical test and generating heatmap or boxplot in this study, bedtools Version 2.19.1 was used for classification of different types of lncRNAs.

For manuscripts utilizing custom algorithms or software that are central to the research but not yet described in published literature, software must be made available to editors/reviewers upon request. We strongly encourage code deposition in a community repository (e.g. GitHub). See the Nature Research guidelines for submitting code & software for further information.

Data

Policy information about availability of data

All manuscripts must include a data availability statement. This statement should provide the following information, where applicable:

- Accession codes, unique identifiers, or web links for publicly available datasets
- A list of figures that have associated raw data
- A description of any restrictions on data availability

RNA-Seq and sRNA-seq datasets generated in this study can be found in the NCBI Gene Expression Omnibus under accession number GSE42695 [https://

www.ncbi.nlm.nih.gov/geo/query/acc.cgi?acc=GSE42695] and GSE120709 [https://www.ncbi.nlm.nih.gov/geo/query/acc.cgi?acc=GSE120709]. The source data underlying Figures 2-6 and Supplementary Figures 2-3, 5-6 and 8-10 are provided as a Source Data file. All other data that support the findings of this study are available from the corresponding author upon request.

Field-specific reporting

Please select the best fit for your research. If you are not sure, read the appropriate sections before making your selection.

☒ Life sciences ☐ Behavioural & social sciences ☐ Ecological, evolutionary & environmental sciences

For a reference copy of the document with all sections, see nature.com/authors/policies/ReportingSummary-flat.pdf

Life sciences study design

All studies must disclose on these points even when the disclosure is negative.

Sample size	The sample sizes are described in the methods and figure legends.
Data exclusions	No data were excluded.
Replication	All attempts at replication were successful.
Randomization	All samples were collected randomly.
Blinding	The experiments were not blinded.

Reporting for specific materials, systems and methods

Materials & experimental systems

n/a	Involved in the study
☐	☒ Unique biological materials
☐	☒ Antibodies
☒	☐ Eukaryotic cell lines
☒	☐ Palaeontology
☒	☐ Animals and other organisms
☒	☐ Human research participants

Methods

n/a	Involved in the study
☒	☐ ChIP-seq
☒	☐ Flow cytometry
☒	☐ MRI-based neuroimaging

Unique biological materials

Policy information about availability of materials

Obtaining unique materials	Correspondence and requests for materials should be addressed to Yijun Qi.

Antibodies

Antibodies used	anti-WDR5 (ab75439, Abcam), anti-FLAG (F1804, Sigma), anti-H3K4me3 (04-745, Millipore), anti-BrdU (Ab1893,Abcam), anti-H3K27ac (ab4729,Abcam), anti-H3K36me3 (ab9050,Abcam), anti-H3K27me3 (07-449, Millipore)
Validation	All the antibodies are available and have been clearly described in the paper.
