# Supplementary material for: Global identification of Arabidopsis lncRNAs reveals the regulation of MAF4 by a natural antisense RNA
Source: Nat Commun. 2018 Nov 29;9:5056. doi: 10.1038/s41467-018-07500-7 (PMC6265284; doi:10.1038/s41467-018-07500-7)
Supplement: Supplementary file 2 — Peer Review [file 41467_2018_7500_MOESM2_ESM.pdf]

Reviewers' Comments:

Reviewer #1:
Remarks to the Author:
This paper describes the characterization of 12,606 lncRNAs in Arabidopsis under normal or stress conditions. As in many other experiments, they found a positive correlation between natural antisense transcripts and their cognate sense genes. They characterized in more detail, one lncRNA, MAS, a NAT lncRNA produced from the MADS AFFECTING FLOWERING4 (MAF4) locus. MAS is required for MAF4 expression through the interaction with WDR5a, one core component of the COMPASS-like complexes. MAS is proposed to recruit WDR5a to MAF4 to enhance histone 3 lysine 4 trimethylation (H3K4me3) in this locus.
The paper is well written and reveals a novel function of a lncRNA in the regulation of flowering. Interestingly, this mechanism is put into perspective with other related mechanisms in animal systems, and this work will be of interest for a very broad audience.
There are some comments that need to be addressed:
a) The Overlapping lncRNA class (OT-lncRNA) class is not really meaningful. Indeed, partial coverage of protein coding transcripts by short reads could lead to the prediction of new lncRNA transcript inside an mRNA and likely further experiments are really required to confirm their "overlapping nature". In addition, wrongly annotated 5' or 3' UTR could also lead to the detection of "new" OT-lncRNA. I suggest that this class is removed from the final analysis or better defined. TO my view this does not change in any point the main message of the paper.
b) The authors claim that "the p.c.c. values of Overlapping NAT-lncRNA/sense gene pairs were significantly higher than the values between adjacent protein coding pairs (Fig. 3a), suggesting that Overlapping NAT-lncRNAs have a stronger tendency to having positively correlated expression patterns with their sense overlapping genes." It was shown that the strand specificity of the dUTP method for RNA-seq is not absolute. Levin et al (Nat Meth. 2010) found that dUTP based SS-RNA-seq lead to about 10% of spurious antisense reads. This implies that NAT annotation should be carefully addressed only based on antisense counts. They should rather include the ratio between mRNA and NAT in a statistical model such as the one described by Li et al., Genome Research 2013 (10.1101/gr.149310.112). Therefore, the large number of positive correlations between mRNAs and NATs should be subjected to caution since it may also be due to the strong induction of mRNA in certain conditions/ stress leading to artificial Ovelapping NATs.
c) Authors claim that "NAT-lncRNAs regulate the expression of cognate sense genes". This broad conclusion is based on the fact the authors found that 15 out of 21 NAT positively regulate their sense mRNA. However, this observation is based on the use of amiRNA targeting of the NAT transcript. Even though miRNAs do not generally produce secondary siRNAs, depending on the size of the molecule produced by the amiRNA (21 or 22), secondary production of siRNA targeting sense mRNA or miRNA* targeting of the mRNA may occur. Small RNA northern blots with sense or antisense RNA probes (or other siRNA detection method) could help to exclude such possibility. Alternatively, amiRNA lines targeting the sense strand could provide a definitive answer to the strand specificity of the amiRNA silencing method.
d) In addition, the strand specificity of qRT-PCR on overlapping transcripts is somewhat difficult to totally exclude spurious reverse transcription. A Northern Blot of mRNA in amiRNA line would clearly reinforce author's conclusions about the NAT-mediated regulation of the sense mRNA. Nevertheless, the use of the inducible promoter for MAS to induce the sense cognate mRNA clearly supports the model. A kinetic of this experiment to show that MAS is induced before MAF4 will be a nice addition to the paper.

Reviewer #2:
Remarks to the Author:
This paper is quite interesting, very readable, and thorough in term of experiments and their analysis. I have therefore only two questions:

1) Regarding the properties of lncRNAs compared to mRNAs: Could the observed differences be due to the difference in expression level? For example for lowly expressed transcripts, RNAseq may not cover the full transcript, resulting in an incomplete assembly. I would suggest to do this comparison on expression-matched sets of lncRNAs and mRNAs.

2) Regarding the knockdown experiments using artificial miRNAs: Is it possible that the passenger strand of the artificial miRNA binds to the sense gene, and cause the reduction in their expression?

Reviewer #3:
Remarks to the Author:
In this manuscript, Zhao et al. used strand-specific RNA sequencing to identify 12,606 lncRNAs in Arabidopsis under normal or stress conditions. Through a series of bioinformatics' analyses, the authors found that the expression of natural antisense transcripts (NATs) lncRNAs often positively correlates with the expression of their cognate sense genes. So, the authors picked up a new NAT-lncRNA named MAS to do the further study. The authors characterized that MAS is induced by cold and is indispensable for the activation of MAF4 transcription and suppression of precocious flowering. Mechanistically, the authors showed that MAS activates MAF4 by interacting with WDR5a, and recruiting WDR5a to MAF4 to enhance histone 3 lysine 4 trimethylation (H3K4me3). Overall, this manuscript expands on our current knowledge regarding how NAT-lncRNAs regulate gene expression in vernalization response and the mechanism underlying the role of MAS-COMPASS-like complex pathway regulation. However, the genome wide identification of lncRNAs in Arabidopsis was initial published in 2012 (PMID: 23136377) and has been reviewed in 2015 (PMID: 25936895). This manuscript focus on NATs, which might provide certain novelty. The following major concerns also need be addressed.
Major concerns:
1. Fig2a, the heat map showed that for the different treated conditions, it seems like they just use one sample to compare to each other. Each group need at least 3 samples.
2. In Fig3a, the p.c.c. values of OTC gene pairs also showed significantly higher values than the values between adjacent protein coding pairs. The authors need to justify the rationale underlying selecting NAT-lncRNAs to further validate?
3. The rationale to select NAT-lncRNA_2962 (MAS) need to be clarified.
4. In Fig4, The authors concluded that MAS regulates the transcription of MAF4, leading to reduced impaired phenotype. The functional role of MAS and the axis of MAS-MAF4 need to be validated by rescue experiments. Please consider addressing whether overexpressed MAF4 in the MAS knockdown lines could rescue the phenotype.
5. MAF5 is also near the MAS gene and also showed a similar expression pattern in the cold treatment condition. The effect of MAS may also regulate the expression of MAF5. The author need to demonstrate whether MAS regulates MAF5 expression and determine whether the altered MAF5 affects vernalization response.
6. To understand the underlying molecular mechanism of MAS-mediated transcriptional regulation of MAF4, RAP assay (PMID: 23828888) or CHIRP assay (PMID: 22472705) need to be applied to indicate the chromatin association of MAS.
7. In Fig6, to conclude that the MAS-mediated recruitment of WDR5A to the MAF4 TSS is not mediated by other cofactors, the authors could provide in vitro RNA-pulldown array using recombinant WDR5 and in vitro transcribed MAS RNA to demonstrate the directness of WDR5 interction.
8. The author need to determine the status of other histone markers for MAF4 transcription activation, such as H3K27Ac.

Point-by-Point Responses:

We appreciate the constructive comments made by the reviewers. We have provided additional data and revised our manuscript to address the concerns raised by the reviewers. We wish the revisions are sufficient and the manuscript is now acceptable for publication. Point-by-point responses are listed below.

Reviewer #1

This paper describes the characterization of 12,606 lncRNAs in Arabidopsis under normal or stress conditions. As in many other experiments, they found a positive correlation between natural antisense transcripts and their cognate sense genes. They characterized in more detail, one lncRNA, MAS, a NAT lncRNA produced from the MADS AFFECTING FLOWERING4 (MAF4) locus. MAS is required for MAF4 expression through the interaction with WDR5a, one core component of the COMPASS-like complexes. MAS is proposed to recruit WDR5a to MAF4 to enhance histone 3 lysine 4 trimethylation (H3K4me3) in this locus.

The paper is well written and reveals a novel function of a lncRNA in the regulation of flowering. Interestingly, this mechanism is put into perspective with other related mechanisms in animal systems, and this work will be of interest for a very broad audience.

There are some comments that need to be addressed:

a) The Overlapping lncRNA class (OT-lncRNA) class is not really meaningful. Indeed, partial coverage of protein coding transcripts by short reads could lead to the prediction of new lncRNA transcript inside an mRNA and likely further experiments are really required to confirm their "overlapping nature". In addition, wrongly annotated 5' or 3' UTR could also lead to the detection of "new" OT-lncRNA. I suggest that this class is removed from the final analysis or better defined. TO my view this does not change in any point the main message of the paper.

Response: We agree with the reviewer's concern and have removed the overlapping lncRNA (OT-lncRNA) class and revised our manuscript accordingly.

b) The authors claim that "the p.c.c. values of Overlapping NAT-lncRNA/sense gene pairs were significantly higher than the values between adjacent protein coding pairs (Fig. 3a), suggesting that Overlapping NAT-lncRNAs have a

stronger tendency to having positively correlated expression patterns with their senseoverlapping genes." It was shown that the strand specificity of the dUTP method for RNA-seq is not absolute. Levin et al (Nat Meth. 2010) found that dUTP based SS-RNA-seq lead to about 10% of spurious antisense reads. This implies that NAT annotation should be carefully addressed only based on antisense counts. They should rather include the ratio between mRNA and NAT in a statistical model such as the one described by Li et al., Genome Research 2013 (10.1101/gr.149310.112). Therefore, the large number of positive correlations between mRNAs and NATs should be subjected to caution since it may also be due to the strong induction of mRNA in certain conditions/ stress leading to artificial Overlapping NATs.

Response: As estimated by RSeQC[1], we found that the percentages of spurious antisense reads in our strand-specific RNA-seq datasets were less than 1%. Thus, there is a low probability that our annotated NAT-lncRNAs come from spurious antisense reads.

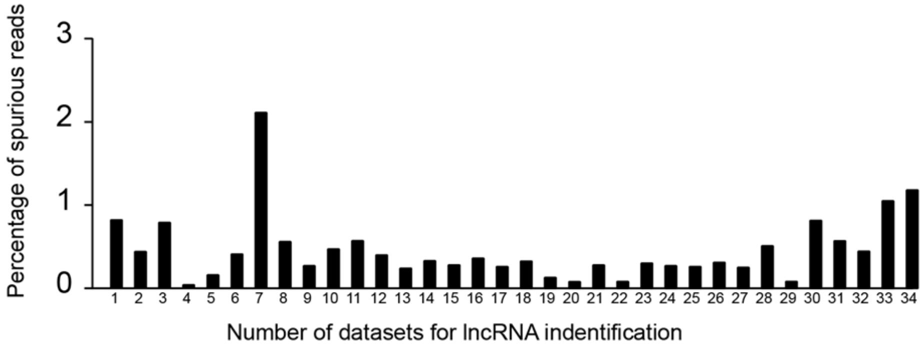

Figure legend: the percentage of spurious antisense reads in RNA-seq datasets.

As suggested, we filtered the annotated NAT-lncRNAs as described in Li et al., 2013. The new results presented below also suggest that overlapping NAT-lncRNAs have a stronger tendency to have positively correlated expression patterns with their sense overlapping genes.

Notably, we found that the Li et al. method filtered out some lowly expressed transcripts. For example, *MAS*, which is expressed at a low level but can be consistently detected in different libraries, was filtered out by this method.

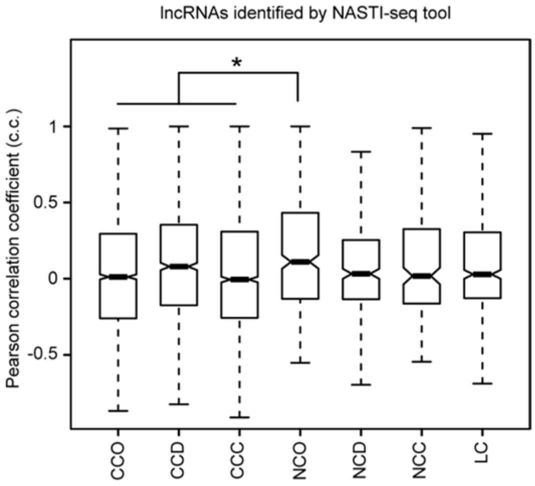

Figure legend: A boxplot showing the correlation of expression patterns between neighboring gene pairs. NAT-lncRNAs are filtered as Li et al., 2013. CCO, overlapped protein-coding gene pair; CCD, divergent protein-coding gene pair; CCC, convergent protein-coding gene pair; NCO, overlapping NAT-lncRNA and associated protein coding gene pair; NCD, divergent NAT-lncRNA and closest neighboring gene pair; NCC, convergent NAT-lncRNA and closest neighboring gene pair; LC, lincRNA and closest neighboring gene pair. Asterisks indicate a significant difference between the indicated groups (Mann–Whitney U test, P value < 0.05).

c) Authors claim that "NAT-lncRNAs regulate the expression of cognate sense genes". This broad conclusion is based on the fact the authors found that 15 out of 21 NAT positively regulate their sense mRNA. However, this observation is based on the use of amiRNA targeting of the NAT transcript. Even though miRNAs do not generally produce secondary siRNAs, depending on the size of the molecule produced by the amiRNA (21 or 22), secondary production of siRNA targeting sense mRNA or miRNA targeting of the mRNA may occur. Small RNA northern blots with sense or antisense RNA probes (or other siRNA detection method) could help to exclude such possibility. Alternatively, amiRNA lines targeting the sense strand could provide a definitive answer to the strand specificity of the amiRNA silencing method.*

Response: We performed small RNA sequencing with two *MAS-amiRNA* lines and twelve other *amiRNA* lines (randomly chosen). Our results revealed that no secondary siRNAs were produced in these lines (Fig. 4a and Supplementary Fig. 7 in the revised manuscript).

We further analyzed the extents of complementarity between amiRNA*s and sense mRNAs in these lines. Eight out of 23 amiRNA*s do not base pair with sense mRNAs at all. The rest of the amiRNA*s have mismatches to corresponding sense mRNAs at critical positions (Supplementary Fig. 4 and

Supplementary Fig. 8b in the revised manuscript), making it less likely that these amiRNA*s target sense mRNAs. Furthermore, most of the amiRNA*s do not have 5' terminal uridine (Supplementary Fig. 4 in the revised manuscript), making it less likely that they are loaded into the effector AGO1 to suppress gene expression[2].

We also added the data showing that knocking down *MAF4* by amiRNAs did not affect the expression of *MAS* (Fig. 4d in the revised manuscript), validating the strand specificity of the amiRNA silencing method.

d) In addition, the strand specificity of qRT-PCR on overlapping transcripts is somewhat difficult to totally exclude spurious reverse transcription. A Northern Blot of mRNA in amiRNA line would clearly reinforce author's conclusions about the NAT-mediated regulation of the sense mRNA. Nevertheless, the use of the inducible promoter for MAS to induce the sense cognate mRNA clearly supports the model. A kinetic of this experiment to show that MAS is induced before MAF4 will be a nice addition to the paper.

Response: For detection of mRNA levels in 16 out of 21 amiRNA lines, we designed primers that do not hybridize the overlapping regions of NAT-lncRNAs and mRNAs (Supplementary Fig. 4). Such design can exclude the problem of spurious reverse transcription. As suggested, we have added the kinetics of *MAS* and *MAF4* induction upon β-estradiol treatment in our revised manuscript (Fig. 5c). *MAS* is immediately induced after β-estrogen treatment, whereas *MAF4* exhibit significant upregulation 12 hours post β-estrogen stimulation, posterior to *MAS*.

Reviewer #2

This paper is quite interesting, very readable, and thorough in term of experiments and their analysis. I have therefore only two questions:

1) Regarding the properties of lncRNAs compared to mRNAs: Could the observed differences be due to the difference in expression level? For example for lowly expressed transcripts, RNAseq may not cover the full transcript, resulting in an incomplete assembly. I would suggest to do this comparison on expression-matched sets of lncRNAs and mRNAs.

Response: We performed the analysis on expression-matched sets of lncRNAs and mRNAs. We presented the results below. Similar to our previous results, the results show that the lncRNAs are much shorter and with fewer exons, whereas the isoform numbers of lowly expressed mRNAs are comparable to that of lncRNAs. Thus, the observed difference of transcript length and exon numbers are not due to the difference in expression levels.

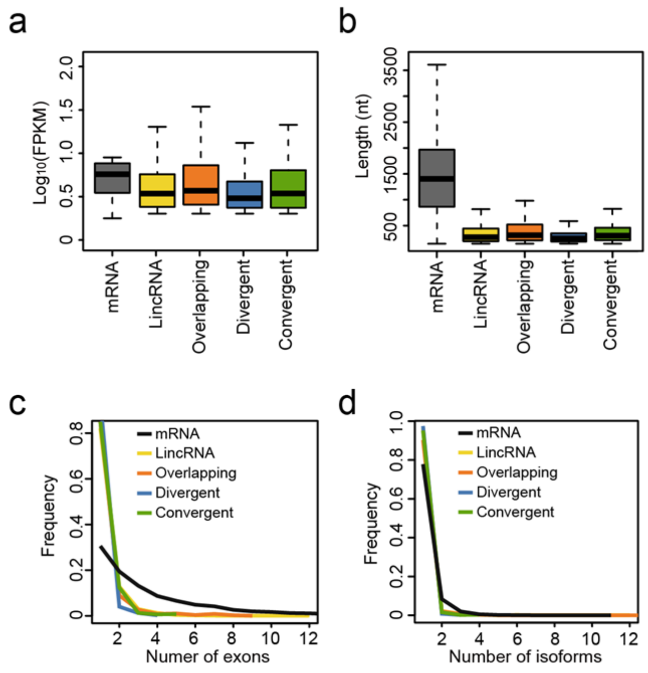

Figure legend: (a) A boxplot shows the expression levels of lncRNAs and mRNA. (b) A boxplot shows the length distribution of lowly expressed mRNA and lncRNAs. (c) A frequncy plot shows the distribution of exon numbers. (d) A frequency plot shows the distribution of isoform numbers.

2) Regarding the knockdown experiments using artificial miRNAs: Is it possible that the passenger strand of the artificial miRNA binds to the sense gene, and cause the reduction in their expression?

Response: Please see our response to the Point c) of Reviewer #1.

Reviewer #3

In this manuscript, Zhao et al. used strand-specific RNA sequencing to identify 12,606 lncRNAs in Arabidopsis under normal or stress conditions. Through a series of bioinformatics' analyses, the authors found that the expression of natural antisense transcripts (NATs) lncRNAs often positively correlates with the expression of their cognate sense genes. So, the authors picked up a new NAT-lncRNA named MAS to do the further study. The authors characterized that MAS is induced by cold and is indispensable for the activation of MAF4 transcription and suppression of precocious flowering. Mechanistically, the authors showed that MAS activates MAF4 by interacting with WDR5a, and recruiting WDR5a to MAF4 to enhance histone 3 lysine 4 trimethylation (H3K4me3).

Overall, this manuscript expands on our current knowledge regarding how NAT-lncRNAs regulate gene expression in vernalization response and the mechanism underlying the role of MAS-COMPASS-like complex pathway regulation. However, the genome wide identification of lncRNAs in Arabidopsis was initial published in 2012 (PMID: 23136377) and has been reviewed in 2015 (PMID: 25936895). This manuscript focus on NATs, which might provide certain novelty. The following major concerns also need be addressed.

Major concerns:

1. Fig2a, the heat map showed that for the different treated conditions, it seems like they just use one sample to compare to each other. Each group need at least 3 samples.

Response: As suggested by the reviewer, we have repeated the analyses and three replicates for each treated condition are now included in our revised manuscript (Fig. 2a and Supplementary Fig. 3).

2. In Fig3a, the p.c.c. values of OTC gene pairs also showed significantly higher values than the values between adjacent protein coding pairs. The authors need to justify the rationale underlying selecting NAT-lncRNAs to further validate?

Response: As also suggested by Reviewer #1, the OT-lncRNA class has been removed in the revised manuscript.

3. The rationale to select NAT-lncRNA_2962 (MAS) need to be clarified.

Response: The finding that NAT-lncRNAs regulates cognate sense gene expression prompted us to investigate the biological importance of such regulation. We focused on *MAS* because it is transcribed from the antisense strand of the cold-responsive *MAF4* gene, a *FLC* family member that is known to function to prevent precocious vernalization response.

4. In Fig4, The authors concluded that MAS regulates the transcription of MAF4, leading to reduced impaired phenotype. The functional role of MAS and the axis of MAS-MAF4 need to be validated by rescue experiments. Please consider addressing whether overexpressed MAF4 in the MAS knockdown lines could rescue the phenotype.

Response: Our Fig. 4d (changed to Fig. 4e in the revised manuscript) and Fig 5b showed that knocking down *MAS* reduces *MAF4* expression. Our Fig. 5d in the revised manuscript showed that the induction of *MAS* induces the transcription of *MAF4*. These data provide strong evidence that *MAS* play a

role in the regulation of *MAF4* expression. *amiR-MAS-1/2* lines show precious flowering phenotype. Because *MAF4* expression is downregulated in these lines and *MAF4* is known to be a floral repressor [3,4], we considered that the early flowering phenotype is due to *MAF4* misregulation. Thus, the lncRNA *MAS*, by regulating the expression of an FLC family member, *MAF4*, fine-tune the time of flowering. A rescue experiment will rule out the possibility that early flowering is caused by other factors and further support our conclusion. However, generating stable transgenic lines overexpressing *MAF4* takes more than 6 months. We discussed with the handling editor and he agrees that the rescue experiment is not essential.

5. MAF5 is also near the MAS gene and also showed a similar expression pattern in the cold treatment condition. The effect of MAS may also regulate the expression of MAF5. The author need to demonstrate whether MAS regulates MAF5 expression and determine whether the altered MAF5 affects vernalization response.

Response: As suggested by the reviewer, we detected *MAF5* expression in *amiR-MAS-1/2* knockdown lines. Our results show that *MAF5* expression was not affected by *MAS* knockdown (Supplementary Fig. 8c in the revised manuscript).

6. To understand the underlying molecular mechanism of MAS-mediated transcriptional regulation of MAF4, RAP assay (PMID: 23828888) or CHIRP assay (PMID: 22472705) need to be applied to indicate the chromatin association of MAS.

Response: As suggested, we performed ChIRP qPCR to detect whether *MAS* associates with the *MAF4* gene. Our results show that *MAS* indeed bound the *MAF4* gene (Fig. 6d in the revised manuscript).

7. In Fig 6, to conclude that the MAS-mediated recruitment of WDR5A to the MAF4 TSS is not mediated by other cofactors, the autors could provide in vitro RNA-pulldown array using recombinant WDR5 and in vitro transcribed MAS RNA to demonstrate the directness of WDR5 interaction.

Response: It has been shown that the human homolog of WDR5A, WDR5, is an RNA binding protein and its recombinant protein binds lncRNA in vitro. Moreover, its binding activity requires a conserved residue F266 [5]. Arabidopsis WDR5A shares very high homology with human WDR5 and possess the conserved residue. Thus, it is most likely that recombinant Arabidopsis WDR5 binds RNAs in vitro. Using RNA IP with Flag-tagged WDR5A or WDR5A(F250A) that is defective in RNA binding, we show that WDR5A binds MAS in vivo and such binding requires the RNA binding activity of WDR5A (Fig.

6e in the revised manuscript). Similarly, using ChIP qPCR, we show that the RNA binding activity of WDR5A is required for its recruitment to *MAF4*. These results strongly indicate that the recruitment of WDR5A to chromatin is mediated by RNA. We further show that *MAS* knockdown compromised WDR5A enrichment at *MAF4* (Fig. 6f in the revised manuscript), suggesting that *MAS* is required for the recruitment of WDR5A to chromatin. We would think that, even if we show that WDR5A binds RNAs in vitro, we still cannot exclude the possibility that recruitment of WDR5A to *MAF4* locus could be also mediated by other factors. We have discussed with the editor and he agrees that the in vitro binding assay is not conclusive either. For this reason, we did not perform the in vitro binding assay.

8. The author need to determine the status of other histone markers for MAF4 transcription activation, such as H3K27Ac.

Response: We detected the levels of H3K27Ac, H3K36me3 and H3K27me3 at the *MAF4* locus in Col-0 and *amiR-MAS-1/2* lines. The levels of H3K27Ac and H3K36me3 remained unaltered while the levels of H3K27me3 were slightly increased in in *amiR-MAS-1/2* lines (Supplementary Fig. 9 in the revised manuscript).

References

1. Wang, L., Wang, S. & Li, W. RSeQC: quality control of RNA-seq experiments. *Bioinformatics* **28**, 2184-5 (2012).
2. Mi, S.J. *et al.* Sorting of small RNAs into Arabidopsis argonaute complexes is directed by the 5 ' terminal nucleotide. *Cell* **133**, 116-127 (2008).
3. Kim, D.H. & Sung, S. Coordination of the Vernalization Response through a VIN3 and FLC Gene Family Regulatory Network in Arabidopsis. *Plant Cell* **25**, 454-469 (2013).
4. Ratcliffe, O.J., Kumimoto, R.W., Wong, B.J. & Riechmann, J.L. Analysis of the Arabidopsis MADS AFFECTING FLOWERING gene family: MAF2 prevents vernalization by short periods of cold. *Plant Cell* **15**, 1159-1169 (2003).
5. Yang, Y.W. *et al.* Essential role of lncRNA binding for WDR5 maintenance of active chromatin and embryonic stem cell pluripotency. *Elife* **3**(2014).

Reviewers' Comments:

Reviewer #1:
Remarks to the Author:
THe resubmitted version of the paper have adequately addressed all my previous concerns. I think it is an excelent story revealing a new mechanism by which an antisense lncRNA controls gene expression.

Reviewer #2:
Remarks to the Author:
The authors have appropriately addressed the issued raised in my review.

Reviewer #3:
Remarks to the Author:
the authors have addressed all of my questions.

REVIEWERS' COMMENTS:

Reviewer #1 (Remarks to the Author):

THe resubmitted version of the paper have adequately addressed all my previous concerns. I think it is an excelent story revealing a new mechanism by which an antisense lncRNA controls gene expression.

Response: We thank the reviewer for his/her comments.

Reviewer #2 (Remarks to the Author):

The authors have appropriately addressed the issued raised in my review.

Response: We thank the reviewer for his/her comments.

Reviewer #3 (Remarks to the Author):

the authors have addressed all of my questions.

Response: We thank the reviewer for his/her comments.
